# Boosting the Stability of FAPbI_3_ Perovskite Nanocrystal Near‐Infrared Light‐Emitting Diodes with Aromatic Ligands and Organic Host Dispersion

**DOI:** 10.1002/smll.202501159

**Published:** 2025-06-06

**Authors:** Haruka Abe, Mizuho Uwano, Ryota Kobayashi, Kohei Narazaki, Takuya Akiyama, Yuta Ito, Daisuke Yokota, Takao Oto, Shotaro Nishitsuji, Ryohei Yamakado, Takayuki Chiba

**Affiliations:** ^1^ Graduate School of Organic Materials Science Yamagata University 4‐3‐16 Jonan Yonezawa Yamagata 992–8510 Japan; ^2^ Graduate School of Science and Engineering Yamagata University 4‐3‐16 Jonan Yonezawa Yamagata 992–8510 Japan

**Keywords:** aromatic ligand exchange, near‐infrared LEDs, operational device lifetime, perovskite nanocrystals, small‐molecule host dispersion

## Abstract

FAPbI_3_ perovskite nanocrystals (NCs) are promising for near‐infrared (NIR) light‐emitting diodes (LEDs) but face challenges in crystal stability and operational device lifetime. Here, it demonstrated that combining 2,2‐diphenylethylamine (DPEA) aromatic ligand exchange with dispersion in π‐conjugated small‐molecule host materials significantly enhances photostability and operational device stability. The DPEA ligand exchange (4.4%) improved compatibility with host materials, and photoluminescence quantum yields of 87.5% (vs 78.4% for neat NC film) by suppressing energy transfer between NCs. Host‐dispersed NC films exhibited uniform domain structures and enhanced photostability under continuous excitation. When applied to NIR‐LEDs, host‐dispersed NC film achieved electroluminescence at 742 nm, a higher spectral radiance of 39.9 W sr^−1^ m^−2^, and an external quantum efficiency of 11.1%. The operational lifetime extended tenfold to 5.2 h, compared to 0.4 h for neat NC‐LEDs. These findings highlight the potential of organic host matrices to overcome critical stability challenges in perovskite optoelectronics.

## Graphical abstract

Aromatic ligand exchange with 2,2‐diphenylethylamine (DPEA) and dispersion in π‐conjugated organic hosts enhance the photostability and device lifetime of near‐infrared FAPbI_3_ perovskite nanocrystal LEDs. This strategy suppresses energy transfer, improves quantum efficiency, and extends operational stability, offering a robust platform for stable perovskite optoelectronic devices.

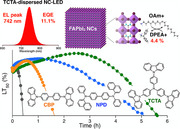

## 1 Introduction

The near‐infrared (NIR) emission spectrum, spanning NIR‐I (650–950 nm) and NIR‐II (1100–1350 nm) ranges, has garnered significant interest due to its applications in optical sensing, communication, bioimaging, data storage, nondestructive testing, photodynamic therapy, and night vision.^[^ ^1^ ^]^ Organic semiconductors, including phosphorescent^[^ ^2^ ^]^ and thermally activated delayed fluorescent emitter, along with colloidal lead chalcogenide quantum dots (QDs,^[^ ^3^ ^]^ have demonstrated suitability for NIR light‐emitting diodes (LEDs) owing to their narrower optical bandgap and broader absorbance ranges. Among these, metal halide perovskites (ABX_3_)—encompassing 3D, quasi‐2D, 3D/2D hybrid, and colloidal nanocrystal (NC) structures—have emerged as promising candidates for NIR‐LEDs due to their tunable optoelectronic properties and solution‐processable fabrication.^[^ ^4^ ^]^ Early research on the NIR‐emitting perovskites demonstrated that quasi‐2D perovskites with multiple quantum‐well structures could achieve high‐efficiency in NIR‐LEDs, attributed to their cascaded energy landscapes facilitating effective electron–hole‐confining.^[^ ^5^, ^6^, ^7^, ^8^, ^9^ ^]^ Additionally, advancements in organic A‐site cation FAPbI_3_ (FA, formamidinium) 3D bulk perovskite, such as surface modification and additive engineering for defect passivation,^[^ ^10^, ^11^, ^12^, ^13^, ^14^, ^15^, ^16^ ^]^ have significantly improved the external quantum efficiency (EQE) of NIR‐LEDs, achieving up to over 20%.^[^ ^17^, ^18^, ^19^ ^]^ However, FAPbI_3_ faces a major challenge: propensity to undergo a phase transition from the emissive α‐phase (α‐FAPbI_3_) to the non‐emissive δ‐phase (δ‐FAPbI_3_) at room temperature, driven by thermodynamic instability.^[^ ^20^, ^21^ ^]^

To overcome this instability, perovskite NCs have been explored, where reduced surface energy in small‐sized crystals improves phase stability.^[^ ^22^, ^23^, ^24^ ^]^ All‐inorganic CsPbX_3_ (X = Cl, Br, I) perovskite NCs exhibit remarkable photoluminescent quantum yield (PLQY) near‐unity^[^ ^25^, ^26^, ^27^ ^]^ and have enabled blue, green, and red LEDs with EQE exceeding 20% through strategies such as surface ligand engineering,^[^ ^28^, ^29^, ^30^, ^31^, ^32^ ^]^ metal doping,^[^ ^33^, ^34^ ^]^ long‐range order NCs,^[^ ^35^, ^36^ ^]^ and optimization of hole/electron transport materials.^[^ ^37^, ^38^ ^]^ However, extending the emission wavelength from red to NIR has been proven challenging. Efforts using A‐site mixed cation compositions, such as Cs_x_FA_1−x_Pb(Br_1−y_I_y_)_3_ NCs, have achieved NIR emission at 735 nm with an EQE (5.9%).^[^ ^39^ ^]^ In contrast, FAPbI_3_ NCs have shown superior NIR‐I emission (740–780 nm) with higher PLQY, effectively overcoming the “Red Wall”.^[^ ^40^, ^41^, ^42^, ^43^ ^]^ Nevertheless, NIR‐LEDs based on FAPbI_3_ NCs suffer from low operational device lifetime (several tens of minutes) compared to 3D bulk FAPbI_3_‐based NIR‐LEDs, which exhibit operational device lifetime of several tens of hours.

The integration of 3D bulk perovskite into organic host matrices offers a promising strategy to address these challenges, as perovskite–molecule composite structure can enhance optical and structural stability while improving device efficiency.^[^ ^44^, ^45^ ^]^ However, this integration is hindered by the poor compatibility between perovskite NCs stabilized with long alkyl ligands and π‐conjugated small molecules or polymers.^[^ ^46^, ^47^ ^]^ This incompatibility often leads to phase separation, non‐uniform NC distribution, and reduced charge transport, ultimately impairing device efficiency. Addressing these limitations requires innovative approaches to improve the chemical interaction between perovskite NCs and host matrices. Recent studies have demonstrated that incorporating hydrogen bonding and van der Waals interactions between π‐conjugated small molecules can suppress non‐radiative recombination and improve LED efficiency.^[^ ^48^, ^49^ ^]^ We previously demonstrated that the A‐site engineering and aromatic ligands could effectively disperse perovskite NCs into organic host matrices, resulting in improved optical and electronic properties.^[^ ^46^, ^47^ ^]^

In this work, we present a synergetic strategy combining 2,2‐diphenylethylamine (DPEA) aromatic ligand exchange on FAPbI_3_ NCs with dispersion into π‐conjugated small‐molecule host materials. By replacing long alkyl ligands with DPEA (4.4% exchange ratio), we achieve enhanced compatibility between FAPbI_3_ NCs and host materials such as di‐1‐naphthyl‐*N*,*N*’‐diphenylbenzidine (NPD), 4,4′,4′'‐Tri‐9‐carbazolyltriphenylamine (TCTA), and 4,4′‐Di(9H‐carbazol‐9‐yl)‐1,1′‐biphenyl (CBP). Importantly, the dispersion of DPEA‐FAPbI_3_ NCs within these small‐molecule hosts lead to the formation of a domain structure with uniform NC size and orientation. This uniformity plays a crucial role in suppressing energy transfer between NCs and mitigating non‐radiative recombination, which directly contributed to improved photostability and operational device stability. Specifically, TCTA‐dispersed DPEA‐FAPbI_3_ NC NIR‐LEDs exhibited an EL at 742 nm with a higher spectral radiance of 39.9 W sr^−1^ m^−2^, an EQE of 11.1%, and an operational device lifetime ten times longer (5.2 h) than that of neat DPEA‐FAPbI_3_ NC‐LEDs. This indicates that the small‐molecule host‐dispersed NCs film is crucial for simultaneously addressing the challenge of photostability and operational device stability.

## 2 Results and Discussion

### 2.1 Synthesis and DPEA Ligand Exchange of FAPbI_3_ NCs

Precisely size‐controlled FAPbI_3_ NCs were synthesized via the hot‐injection method using formamidine acetate, oleic acid (OA), oleylamine (OAm), lead(II) iodide (PbI_2_), and zinc(II) iodide (ZnI_2_) as precursors. The FAPbI_3_ NC size was finely tuned by adjusting the ZnI_2_ concentration at a relatively low reaction temperature (80 °C)^[^ ^50^ ^]^ (Figure S1a, Supporting Information). ZnI_2_‐treated FAPbI_3_ NCs exhibited a reduced average particle size (10.1 nm) compared to untreated FAPbI_3_ NCs (10.6 nm), accompanied by a slight blue shift in the absorption band edge (1.60 eV vs 1.63 eV), as shown in Figure S1b–d (Supporting Information). The PLQY improved significantly from 31.3% to 77.8, with NIR emission blue‐shifting from 756  to 744 nm (Figure S1e, Supporting Information). Stability tests revealed that ZnI_2_‐treated FAPbI_3_ NCs maintained their PL characteristics and crystal structure after one week of air exposure, whereas untreated NCs showed red shifts of PL spectra and sharp XRD peaks, indicative NC aggregation (Figure S2, Supporting Information). These results demonstrate that ZnI_2_ treatment not only enhances size control and defect passivation but also effectively suppresses NC aggregation, thereby improving stability. To further enhance the performance and compatibility of the ZnI_2_‐treated FAPbI_3_ NCs with organic host matrices, a post‐synthetic aromatic DPEA ligand exchange was performed during the reprecipitation purification process (Figure S3, Supporting Information). DPEA was dissolved in dimethyl carbonate (DMC) anti‐solvent and added to the ZnI_2_‐treated FAPbI_3_ NCs dispersed in toluene, followed by stirring for 5 min. After centrifugation, DPEA‐FAPbI_3_ NCs were collected. Unlike ZnI_2_‐treated FAPbI_3_ NCs without DPEA (FAPbI_3_ NCs), which exhibited poor storage stability and reprecipitation within one day, the ZnI_2_‐treated FAPbI_3_ NCs with DPEA (DPEA‐FAPbI_3_ NCs) maintained a stable dispersion without aggregation for over two weeks (Figure S4, Supporting Information).

To gain deeper insight into the role of ZnI₂, we carried out X‐ray photoelectron spectroscopy (XPS) on both untreated and ZnI₂‐treated FAPbI₃ NCs. No Zn *2p* signals were observed in the treated NCs (Figure S7 and Table S1, Supporting Information), confirming that Zn^2^⁺ does not incorporate into the nanocrystal lattice or surface. However, the I/Pb atomic ratio increased significantly, reaching a value close to the ideal stoichiometry of 3.0, suggesting more complete surface passivation. This enhancement is likely due to the transient interaction of Zn^2^⁺ with halide ions during synthesis, improving local iodide availability and modulating nucleation and growth kinetics.^[^ ^50^ ^]^ Notably, this I/Pb ratio remained unchanged after ligand exchange with DPEA, indicating that the improved halide stoichiometry originates from ZnI₂ treatment rather than surface ligand composition. These findings support the hypothesis that Zn^2^⁺ acts as a dynamic, non‐incorporated modulator that facilitates defect passivation and size control without altering the final composition.

### 2.2 Surface and Structural Characterization of DPEA‐FAPbI_3_ NCs

**Figure 1:**
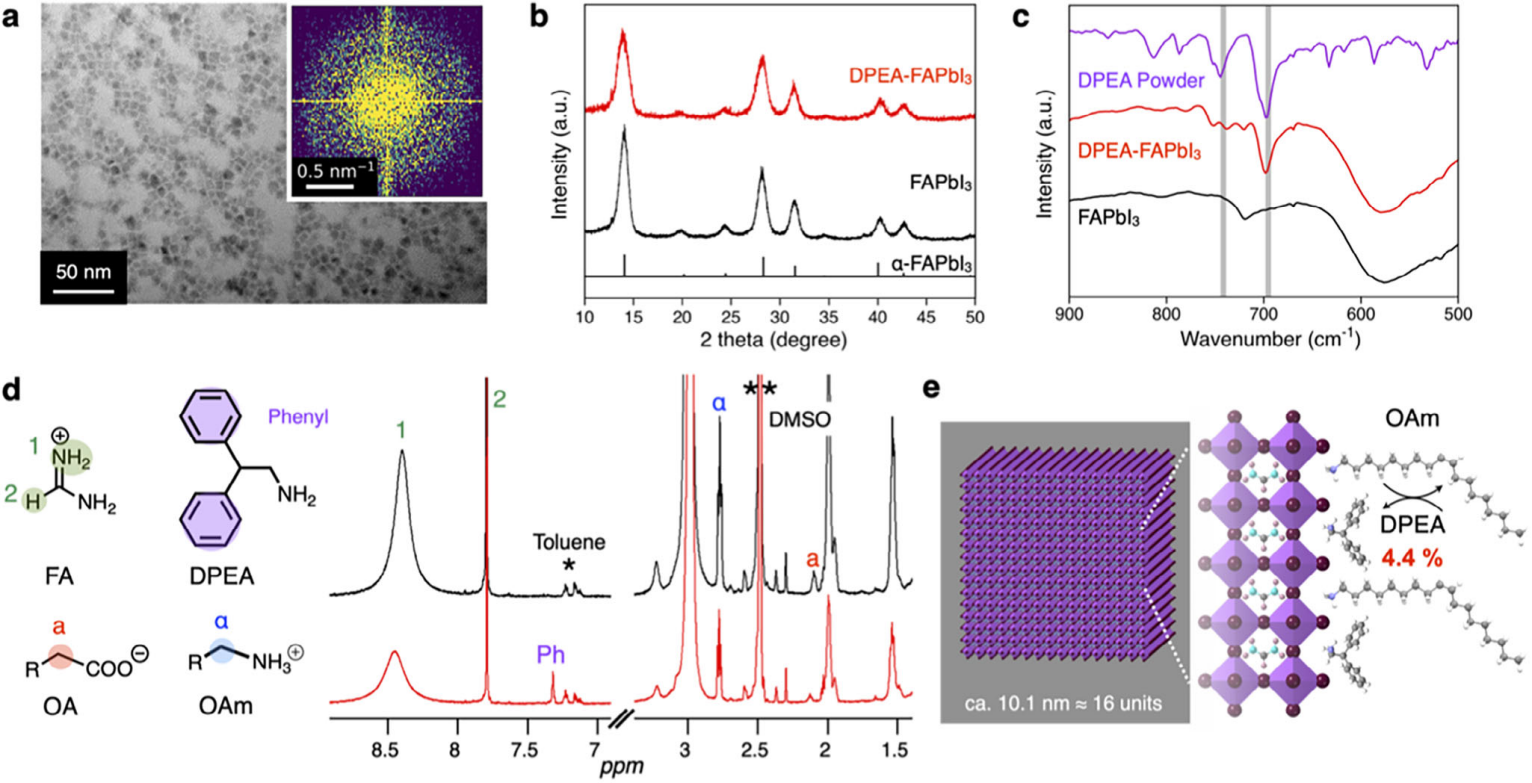
Synthesis and DPEA ligand exchange of FAPbI_3_ NCs. a). TEM image of DPEA‐FAPbI_3_ NCs. b). XRD patterns, c). FTIR, d). ^1^H NMR spectra (in DMSO‐*d_6_*) of the FAPbI_3_ NCs and DPEA‐FAPbI_3_ NCs (*Toluene, **DMSO). e). Calculated surface ligand composition before and after DPEA ligand exchange of FAPbI_3_ NCs.

Transmission electron microscopy (TEM) images confirmed the cubic shape of the DPEA‐FAPbI_3_ NCs with an average size of 10.1 nm, comparable to FAPbI_3_ NCs before ligand exchange (**Figure** **1**a). The fast Fourier transform (FFT) image shows a halo‐like structure in 0.6‐0.9 nm^−1^, which can be attributed to the spatial separation between the NCs (inset in Figure 1a). X‐ray diffraction (XRD) analysis revealed diffraction peaks corresponding to the cubic phase of α‐FAPbI_3_ in both samples, indicating that the DPEA ligand exchange treatment does not alter the NC crystal structures (Figure 1b). Fourier transform infrared (FTIR) spectroscopy identified aromatic mono‐substituents at 752 and 699 cm^−1^ for the DPEA‐FAPbI_3_ NCs, confirming successful ligand attachment (Figure 1c). Compositions analysis using proton nuclear magnetic resonance (^1^H NMR) was performed in DMSO‐*d_6_* (8.1 mg mL^−1^) with ferrocene as an internal standard (0.94 mg mL^−1^) at 100 °C. A signal attributed to the phenyl group of DPEA was detected at 7.3–7.4 ppm in the DPEA‐FAPbI_3_ NCs (Figure 1d; Figure S5, Supporting Information). The molar ratios of FA, OA, OAm, and DPEA relative to ferrocene were 1.43:0.07:0.55 for FAPbI_3_ NCs and 1.21:0.05:0.41:0.02 for DPEA‐FAPbI_3_ NCs, respectively (Table S2, Supporting Information).

These results indicate that DPEA successfully binds to the surface of FAPbI₃ NCs through a ligand exchange process. Although DPEA itself is a neutral amine (−NH₂), we propose that it undergoes proton transfer from surface‐bound oleylammonium (OAm⁺) cations, forming diphenylethylammonium (DPEA⁺). The positively charged DPEA⁺ can then electrostatically bind to the A‐site or interact with the negatively charged surface of the NCs, effectively replacing OAm⁺. This proposed exchange mechanism is supported by previous reports on amine/ammonium pair systems and explains the anchoring behavior of DPEA despite its initial neutral state.^[^ ^51^ ^]^ A schematic illustration of this mechanism is provided in Figure S6 (Supporting Information). Interestingly, calculations based on TEM and NMR indicate that 4.4% of the FAPbI_3_ NC surface are occupied by DPEA (Figure 1e).

Optical characterization using UV–vis absorption, PL spectra, and transient PL measurements revealed that the DPEA‐FAPbI_3_ NC film retained optical properties comparable to those of FAPbI_3_ NCs film (Figure S8, Supporting Information). The DPEA‐FAPbI_3_ NC film exhibited a NIR emission wavelength of 744 nm, with full‐width at half maximum (FWHM) of 45 nm and optical bandgap of 1.66 eV, consistent with the FAPbI_3_ NCs film (excitation wavelength 400 nm). Moreover, the DPEA‐NC film exhibited a high PLQY of 78.4%, with a transient PL decay time of 11.3 ns, closely matching the FAPbI_3_ NCs film (77.8% and 11.0 ns). These results confirm that the ligand exchange process does not negatively impact the intrinsic optical properties of the NCs.

### 2.3 Dispersion of DPEA‐FAPbI_3_ NCs in π‐Conjugated Organic Host Matrices

**Figure 2:**
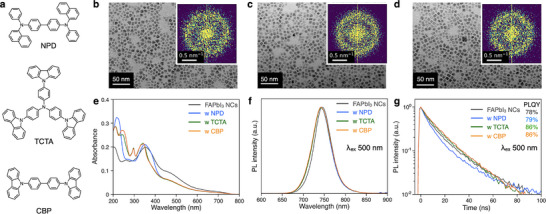
Dispersion of DPEA‐FAPbI_3_ NCs in π‐conjugated organic host matrices. a). Chemical structure of host materials. b–d). TEM images of organic host‐dispersed (NPD, TCTA, and CBP) DPEA‐FAPbI_3_ NCs. e). UV–vis absorption spectra, f). PL spectra, and g transient PL decay lifetime of the DPEA‐FAPbI_3_ NC and organic host‐dispersed NCs.

To investigate the dispersion of DPEA‐FAPbI_3_ NCs into π‐conjugated small‐molecule materials, NPD, TCTA, and CBP were selected as the host matrices (**Figure** **2**a) due to their solubility in toluene, favorable energy level alignment, and compatibility with solution processed film formation. Although perovskite NCs capped with long alkyl ligands typically suffer from poor colloidal stability in aromatic solvents and aggregation issues when mixed with small‐molecule materials, DPEA‐FAPbI_3_ NCs exhibited remarkable colloidal stability in toluene, attributed to the aromatic DPEA ligand, which effectively inhibits the packing of long alkyl chains. Host‐dispersed DPEA‐FAPbI_3_ NC films were successfully prepared by mixing DPEA‐FAPbI_3_ NCs (20 mg mL^−1^ in toluene) with small‐molecule materials (4 mg mL^−1^ in toluene) in a 3:7 volume ratio (Figure S9, Supporting Information). The particle size of host‐dispersed NCs was measured at 9.7 nm for NPD, 10.0 nm for TCTA, and 10.1 nm for CBP, comparable to the DPEA‐FAPbI_3_ NCs without a host matrix (Figure 2b–d). In the FFT images obtained from each TEM image, a clear ring structure at 0.6 nm^−1^ can be seen, indicating that NCs having uniform geometric structure could be successfully prepared (inset in Figure 2b–d).

UV–vis absorption spectra of the host‐dispersed NC films exhibited increased absorbance in the 200–300 nm range, characteristics of the small‐molecule materials (Figure 2e; Figure S10, Supporting Information). Notably, PL spectra of the host‐dispersed NC films exhibited a slight blue shift in emission peak and broader FWHM compared to the neat DPEA‐FAPbI_3_ NC film, which can be attributed to suppressed energy transfer between NCs (Figure 2f; Figure S11, Supporting Information). The host‐dispersed NC films also exhibited enhanced PLQY values of 81.7% for NPD, 87.5% for TCTA, and 86.3% for CBP, indicating effective suppression of concentration quenching. Transient PL decay measurements revealed a slightly increased decay time of 12.1 ns for NPD, 15.6 ns for TCTA, and 14.3 ns for CBP, compared to the neat DPEA‐FAPbI_3_ NC film, highlighting the role of host matrices in stabilizing excited‐state dynamics (Figure 2g; Table S3, Supporting Information).

### 2.4 Enhanced Photostability and Uniform Spatial Distribution of Host‐Dispersed NCs

**Figure 3:**
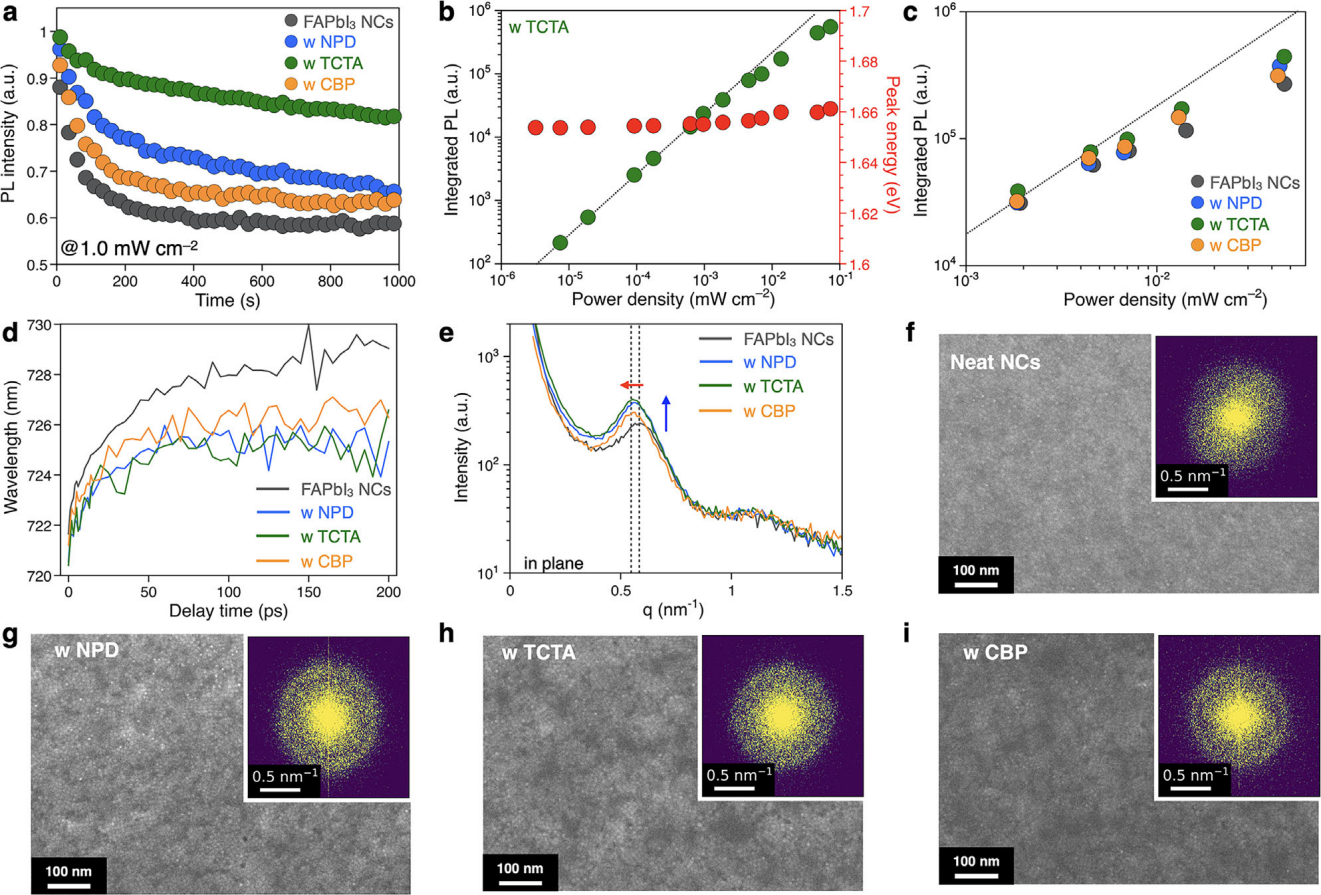
Photostability and structural organic host‐dispersed NCs. Photostability test of under UV irradiation with irradiance. a). PL decay under power density of 1.0 mW cm^−2^ with an excitation wavelength of 405 nm, b). Power‐dependent integrated PL and peak energy shift of TCTA‐dispersed NC film, c). Power‐dependent integrated PL integrated PL of neat and host‐dispersed films. d). Femtosecond transient absorption, e). GISAXS profiles of organic host‐dispersed NC films. g–i). SEM and FFT images (insets) of f). neat NC film, g NPD‐, h TCTA‐, and i CBP‐dispersed NC films.

Photostability tests were conducted on the PL spectra of host‐dispersed NC films under a 405 nm excitation wavelength. The DPEA‐FAPbI_3_ NC film without a host matrix showed a substantial decrease in PL intensity when subjected to an excitation power density of 1.0 mW cm^−2^ (**Figure** **3**a; Figure S12, Supporting Information). In contrast, the TCTA‐dispersed NC film retained 80% of its PL intensity even after 1000 s of continuous irradiation under the same condition, demonstrating the effectiveness of the host‐dispersed strategy. Moreover, the TCTA‐dispersed NC film maintained consistent peak wavelengths and FWHM values across a range of excitation power densities (Figure 3b). The spectrally‐integrated PL intensity of the host‐dispersed NC films displayed a linear increase at low excitation power intensity range (10^−5^ to 10^−3^ mW cm^−2^), transitioning to a nonlinear relationship at higher power intensity (10^−2^ to 10^−1^ mW cm^−2^) (Figure 3c). That nonlinearity implies that inter‐NC carrier interaction in the excited states modulates the photostability. Notably, the emission peak energy of the TCTA‐dispersed NC film remained stable over the entire power ranges, further confirming its enhanced photostability compared to the neat DPEA‐FAPbI_3_ NC film as well as the NPD‐, and CBP‐dispersed film (Figure S13, Supporting Information).

To further investigate the relationship between photoexcited carrier dynamics and photostability, femtosecond transient absorption measurements were performed on the host‐dispersed NC films (Figure 3d; Figure S14, Supporting Information). Within several tens of picoseconds, the bleaching peak of the neat DPEA‐FAPbI_3_ NC films exhibited a large red shift, while a smaller red‐shift was observed for the host‐dispersed NC films. This shift indicates excited state carrier migration among NCs processing different bandgaps, i.e., lock of the NC size and orientation. The results tell that the incorporation of small‐molecule materials effectively suppresses energy transfer between NCs, consistent with observation from the PL spectra.

Grazing incidence small‐angle X‐ray scattering (GISAXS) analysis was conducted to examine the spatial distribution of DPEA‐FAPbI_3_ NCs within various organic host matrices (Figure 3e). In the host‐dispersed films, peaks near a q‐value of 0.6, corresponding to a structure periodicity of ≈10 nm, shifted to lower q‐values relative to the neat DPEA‐FAPbI_3_ NC film, suggesting an increased interparticle distance. Additionally, the host‐dispersed films exhibited increased peak intensity and narrower FWHM, indicative of enhanced structural regularity and alignment of the DPEA‐FAPbI_3_ NCs. These results highlight the role of the host materials (NPD, TCTA, CBP) in promoting a more uniform spatial distribution and better alignment of the NCs.

Scanning electron microscopy (SEM) and FFT analyses further corroborated these findings (Figure 3f–i). The SEM images revealed well‐dispersed NCs with a particle size of ≈10 nm, consistent with the GISAXS results. Corresponding FFT images exhibited characteristic diffraction rings, reflecting the presence of ordered DPEA‐FAPbI_3_ NC arrangements (inset in Figure 3f–i). Among the host materials, the carbazole‐based bulky starburst‐shape TCTA demonstrated minimal interaction with the aromatic DPEA ligand at the NC interface, enabling uniform DPEA‐FAPbI_3_ NC dispersion while preserving crystallinity. This balance optimized both the spatial distribution and the degree of crystallization in the host‐dispersed NC films. The 1D profile derived from the FFT images was in excellent agreement with the GISAXS results, establishing a strong correlation between the real‐space and reciprocal‐space analyses (Figure S15, Supporting Information). To access surface morphology, atomic force microscopy (AFM) measurements were performed on the host‐dispersed NC films. The root mean square roughness (rms) values were 3.40 nm for neat NC film, 2.69 nm for NPD, 2.58 nm for TCTA, and 2.27 nm for CBP (Figure S16, Supporting Information).

### 2.5 Enhanced Operational Stability of Host‐Dispersed DPEA‐FAPbI_3_ NC NIR‐LEDs

**Figure 4:**
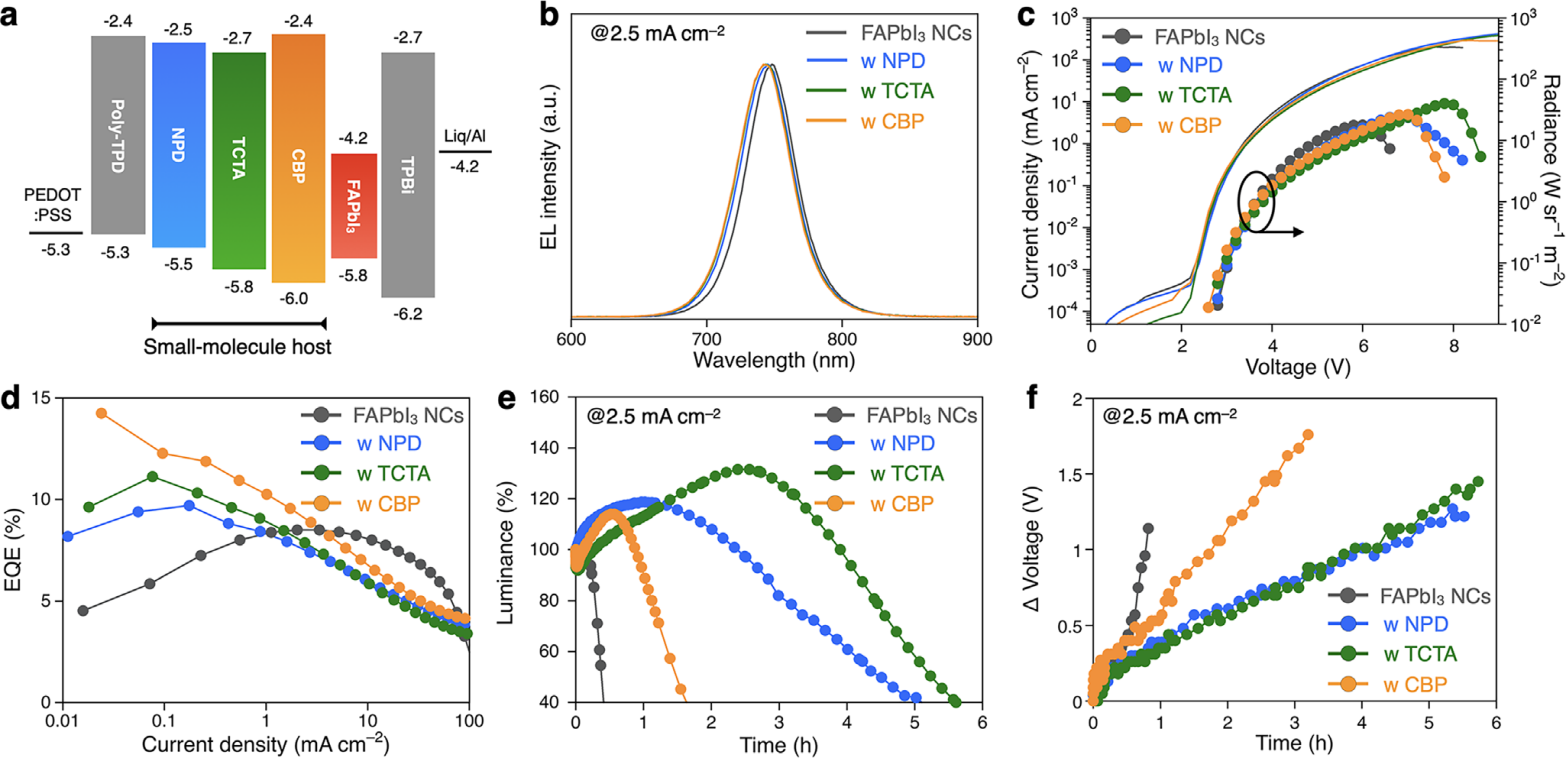
Device characterization of organic host‐dispersed DPEA‐FAPbI_3_ NC‐LEDs. a). Energy diagram of the LEDs incorporating DPEA‐FAPbI_3_ NCs with various organic host materials (NPD, TCTA, and CBP), b). EL spectra, c). current density–voltage–radiance, d). EQE–current density, e). operational device lifetime, and f). driving voltage shift at a constant current density of 2.5 mA cm^−2^.

The DPEA‐FAPbI_3_ NC‐LEDs were fabricated with the following device structure: ITO (130 nm)/poly(3,4‐ethylenedioxythiophene):poly(styrene‐sulfonate) (PEDOT:PSS) with Nafion (40 nm)/poly(4‐butylphenyl‐diphenyl‐amine) (poly‐TPD) (20 nm)/host‐dispersed NCs/tris(1‐phenyl‐1H‐benzimidazole) (TPBi) (50 nm)/Liq (1 nm)/Al (100 nm), as illustrated in **Figure** **4**a. The energy level of the host‐dispersed films was estimated using ultraviolet photoelectron spectroscopy (UPS) (Figure S17, Supporting Information). These LEDs exhibited a consistent NIR emission wavelength of 742 nm, which is slightly blue‐shifted compared to the neat NC‐LED (746 nm), consistent with the PL spectra results (Figure 4b; Figure S18, Supporting Information). The host‐dispersed NC‐LEDs exhibited comparable current density to the neat NC‐LED, indicating that the incorporation of small‐molecule materials (NPD, TCTA, and CBP) does not hinder hole injection and transport due to the deeper ionization potential levels. However, the host‐dispersed NC‐LEDs exhibited enhanced spectral radiance: 24.7 W sr^−1^ m^−2^ for NPD, 39.9 W sr^−1^ m^−2^ for TCTA, and 26.3 W sr^−1^ m^−2^ for CBP, compared to 17.7 W sr^−1^ m^−2^ for the neat NC‐LED (Figure 4c). The EQE–current density characteristics (Figure 4d) reveal that the maximum EQEs were 9.6% for NPD, 11.1% for TCTA, and 14.2% for CBP, all of which surpassed the neat NC‐LED (8.5%). Operational device lifetime tests at a constant current density 2.5 mA cm^−2^, corresponding to an initial spectral radiance of 1.2 W sr^−1^ m^−2^, demonstrated significant stability improvements for the host‐dispersed NC‐LEDs (Figure 4e,f). The LT_50_ (time to 50% of initial brightness) values were 4.5 h for NPD, 5.2 h for TCTA, and 1.5 h for CBP, compared to 0.4 h for the neat NC‐LEDs. These results indicated that the host‐dispersed NC‐LEDs achieve one order of magnitude higher spectral radiance and significantly longer operational device lifetime compared to previously reported FAPbI_3_ NCs based NIR‐LEDs (Tables S4 and S5, Supporting Information). Interestingly, the TCTA‐dispersed NC‐LEDs exhibited a minimal voltage shift during operation, attributed to the suppression of DPEA‐FAPbI_3_ NC degradation, consistent with the photostability tests discussed earlier. These findings demonstrate that the incorporation of a small‐molecule host matrix significantly enhances both photostability and operational stability in perovskite NC‐LEDs. In particular, the carbazole‐based starburst‐shaped TCTA emerged as the most effective host material, enabling uniform NC dispersion, and minimal degradation over time. This study highlights the potential of small‐molecule host‐dispersed strategies in advancing perovskite‐based optoelectronic devices. These improvements can be further rationalized from the charge carrier aspect, where the incorporation of host matrices enhances charge injection balance and suppresses non‐radiative recombination by reducing exciton–exciton annihilation and facilitating spatial separation of NCs. Such mechanisms are consistent with recent reports that underscore the importance of interfacial engineering and charge carrier dynamics in optimizing perovskite based‐LED performance.^[^ ^52^, ^53^ ^]^

## 3 Conclusion

In conclusion, our study demonstrates a significant enhancement in the photostability and operational device lifetime of FAPbI_3_ NCs achieved through the combined effects of aromatic DPEA ligand exchange and dispersion within π‐conjugated small‐molecule host materials. The incorporation of DPEA‐FAPbI_3_ NCs into host matrices such as TCTA, NPD, and CBP resulted in improved compatibility, uniform dispersion, and enhanced structural organization, which collectively contributed to elevated PLQYs and robust EL performance in NIR‐LEDs. Notably, the TCTA‐dispersed NCs exhibited an impressive EQE of 11.1% and a tenfold increase in operational stability compared to neat NC‐based devices. These findings underscore the critical role of organic host matrices in optimizing the optoelectronic properties of perovskite‐based devices, paving the way for more stable and efficient next‐generation light‐emitting technologies.

## 4 Experimental Section

### Materials

Lead iodide (II) (PbI_2_, 99.99%), 2,2‐diphenylethylamin (DPEA, 98.0%), and dimethyl carbonate (DMC, 98.0%) were purchased from TCI. Toluene (99.5%) was purchased from KANTO CHEMICAL. Octadecene (ODE, 90%), oleic acid (OA, 90%), and oleylamine (OAm, 90%), zinc iodide (ZnI_2_, 98%) were obtained from Sigma–Aldrich. PEDOT:PSS was supplied by Clevious, while poly‐TPD was obtained from American Dye Source. TPBi, Liq, NPD, TCTA, and CBP were purchased from e‐Ray Optoelectronics Technology Co. All other chemicals and solvents were purchased from Sigma‐Aldrich and used as received, without further purification.

### Synthesis of FAPbI_3_ NCs

FAPbI_3_ NCs were synthesized by combining PbI_2_ (917 mg) and ZnI_2_ (228 mg) with ODE (53 mL), OA (13.3 mL), and OAm (6.6 mL) in a three‐neck flask. The mixture was heated to 120 °C under vacuum unity fully dissolved. FA‐oleate solution (15 mL), prepared separately, was quickly injected into the precursor solution at 80 °C under a nitrogen atmosphere. After a brief reaction, the mixture was rapidly cooled in an ice‐water bath to room temperature to isolate the FAPbI_3_ NCs.

### DPEA Ligand Exchange and Purification

DPEA (0.1 mmol) was dissolved in 1 mL of DMC. An equal volume of this DPEA solution was added to the synthesized FAPbI_3_ NCs. After mixing, the supernatant was removed by centrifugation at 9000 rpm for 3 min. The precipitate was redispersed in toluene and centrifuged under the same condition. Prior to use, the supernatant was filtered through a PTFE filter.

### Surface and Chemical Composition Characterization

^1^H NMR spectra were recorded on a JEOL ECZ‐600 spectrometer operating at a ^1^H frequency of 600 MHz, and FTIR spectra were measured using a HORIBA IRAffinity‐1S instrument. XPS and UPS were performed using a Thermo Fisher Scientific Theta probe with a He I UV source (21.22 eV) under a high vacuum of≈10^−6^ Pa. Surface roughness was analyzed via an Agilent 5500 SPM in tapping mode.

### Photoluminescence Characterization

UV–vis absorption spectra were measured with a Shimadzu UV‐3150 UV–vis–NIR spectrophotometer, while PL spectra were recorded with a HORIBA FluoroMax Plus luminescence spectrometer. The PLQY was determined using a Hamamatsu C9920–01 integral sphere system. Photostability tests were performed using a micro‐PL system based on an Olympus BX53F2 microscope and a Hamamatsu PMA‐12 photonic multichannel analyzer. An Olympus USH‐1030L mercury lamp, combined with a U‐FVN mirror unit for violet excitation (405 nm), was used as the excitation light source.

### Transient Absorption Measurement

Transient absorption spectra were measured using a pump‐probe spectroscopy system (Helios, Ultrafast system) combined with a Ti:sapphire laser system (Spitfire, Spectra Physics), whose fundamental output (800 nm, 100 fs, 1 kHz) was used to generate two beams, one frequency‐doubled for the pump beam and the other converted to a white‐light continuum for the probe beam.

### Spatial Distribution Characterization

TEM images were captured using a JEOL JEM‐2100F instrument. SEM imaging was conducted using a JEOL JSM‐7600F system operating at 15 kV. XRD patterns were measured by using a Rigaku SmartLab diffractometer. GISAXS measurements were performed using a Rigaku NANO‐Viewer (X‐ray wavelength, 1.54 Å).

### Fabrication of Perovskite NC‐LEDs

ITO substrates were cleaned via ultrasonic spin cleaning and treated with UV–ozone treatment for 10 min. A PEDOT:PSS blend with Nafion (40 nm thick) was spin‐coated onto the ITO at 2,000 rpm for 30 s and annealed at 150 °C for 10 min. Poly‐TPD (8 mg mL^−1^ in chlorobenzene) was spin‐coated onto PEDOT:PSS:Nafion layer and annealed at 100 °C for 10 min. DPEA‐FAPbI_3_ NCs (10 mg mL^−1^ in toluene) or organic host‐dispersed NCs (NPD, TCTA, CBP) were spin‐coated onto a poly‐TPD layer. Subsequently, TPBi (50 nm), Liq (1 nm), and Al (100 nm) were sequentially deposited via thermal evaporation. The resulting NC‐LEDs, with an active area of 4 mm^2^, were encapsulated with epoxy glue and glass covers in an N_2_‐filled glovebox. EL spectra, current density versus voltage and radiance versus voltage characteristics were recorded using a Keithley source unit 2400 and an Otsuka Electronics high sensitive spectroradiometer HS‐1000.

## Conflict of Interest

The authors declare no conflict of interest.

## Author Contributions

T.C. conceived and designed the experiments. H.A., M.U., and T.A. synthesized and purified FAPbI_3_ NCs, performed ligand exchange, and carried out NMR and FTIR measurements. H.A., M.U., R.K., T.A., R.Y., and T.C. conducted UV–vis absorption, PL, transient PL, and NMR analyses. M.U., K.N., and Y.I. performed XPS, SEM, and TEM measurements. H.A. and S.N. carried out GISAXS measurements, while Y.I. also contributed to transient absorption measurements. D.Y. and T.O. conducted photostability tests. M.U. and T.C. fabricated the LED devices. T.C. assisted with material and device characterization and wrote the manuscript. All authors reviewed and commented on the manuscript.

## Supporting information



Supporting Information

## Data Availability

The data that support the findings of this study are available in the supplementary material of this article.
