# Supplementary material for: Boosting the Stability of FAPbI3 Perovskite Nanocrystal Near‐Infrared Light‐Emitting Diodes with Aromatic Ligands and Organic Host Dispersion
Source: Small. 2025 Jun 6;21(31):2501159. doi: 10.1002/smll.202501159 (PMC12332826; doi:10.1002/smll.202501159)
Supplement: Supplementary file 1 — Supporting Information [file SMLL-21-2501159-s001.docx]

Supporting Information

**Boosting the Stability of FAPbI3 Perovskite Nanocrystal Near-Infrared Light-Emitting Diodes with Aromatic Ligands and Organic Host Dispersion**

*Haruka Abe, Mizuho Uwano, Ryota Kobayashi, Kohei Narazaki, Takuya Akiyama, Yuta Ito,* *Daisuke Yokota, Takao Oto, Shotaro Nishitsuji, Ryohei Yamakado, Takayuki Chiba^*^*

*Haruka Abe, Mizuho Uwano, Ryota Kobayashi, Kohei Narazaki, Takuya Akiyama, Yuta Ito,* *, Shotaro Nishitsuji, Ryohei Yamakado, Takayuki Chiba*

Graduate School of Organic Materials Science, Yamagata University, 4-3-16 Jonan, Yonezawa, Yamagata 992-8510, Japan

*Daisuke Yokota, Takao Oto*

Graduate School of Science and Engineering, Yamagata University, 4-3-16 Jonan, Yonezawa, Yamagata 992-8510, Japan

E-mail: [T-chiba@yz.yamagata-u.ac.jp](mailto:T-chiba@yz.yamagata-u.ac.jp)


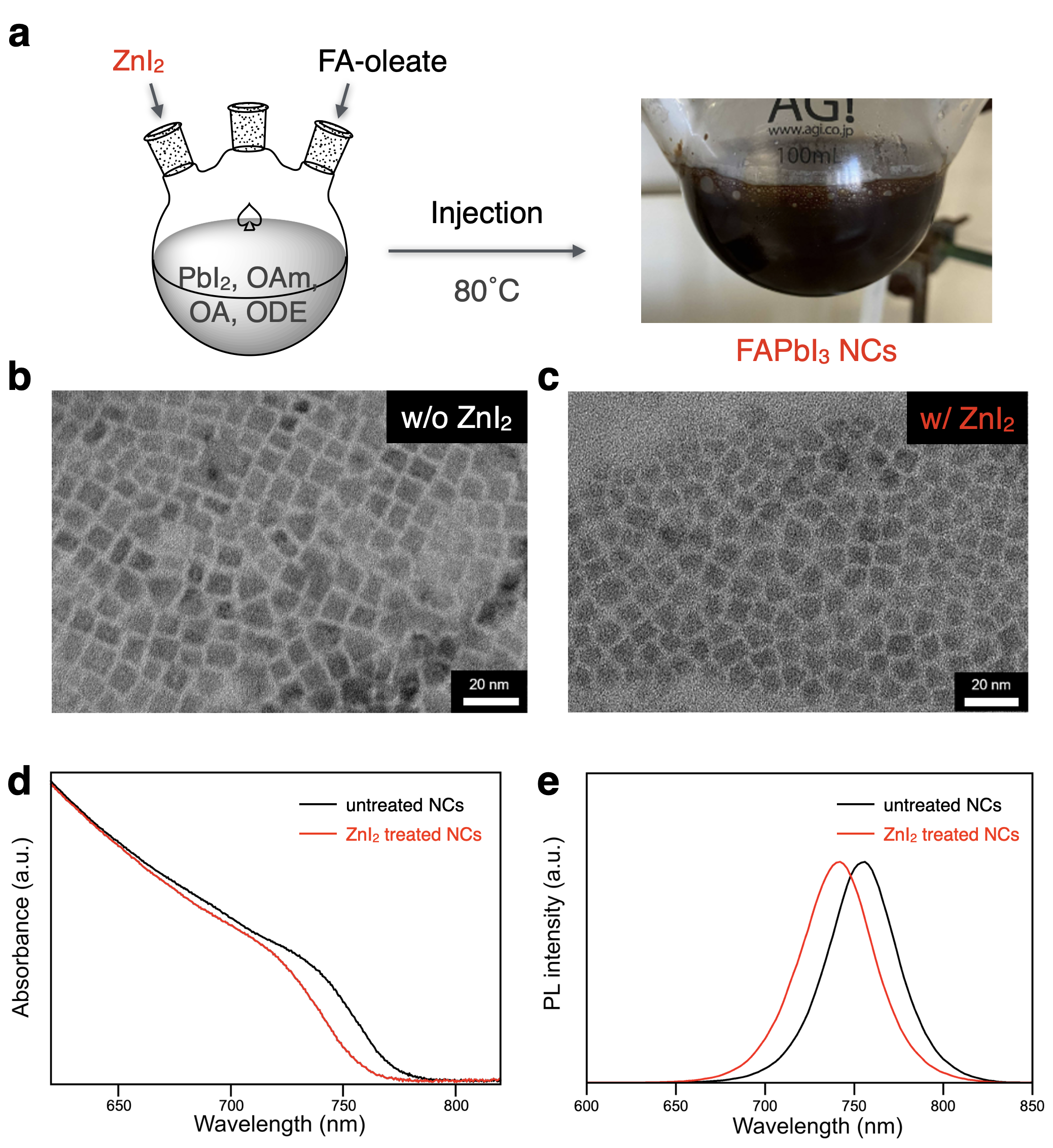


**Figure S1** **Synthesis and characterization of FAPbI_3_ NCs with and without ZnI_2_-treatment.** (a) Schematic diagram of the synthesis process. TEM images of (b) FAPbI_3_ NCs without ZnI_2_-treatment and (c) FAPbI_3_ NCs with ZnI_2_-treatment. (d) UV-vis absorption, (e) PL spectra.

**
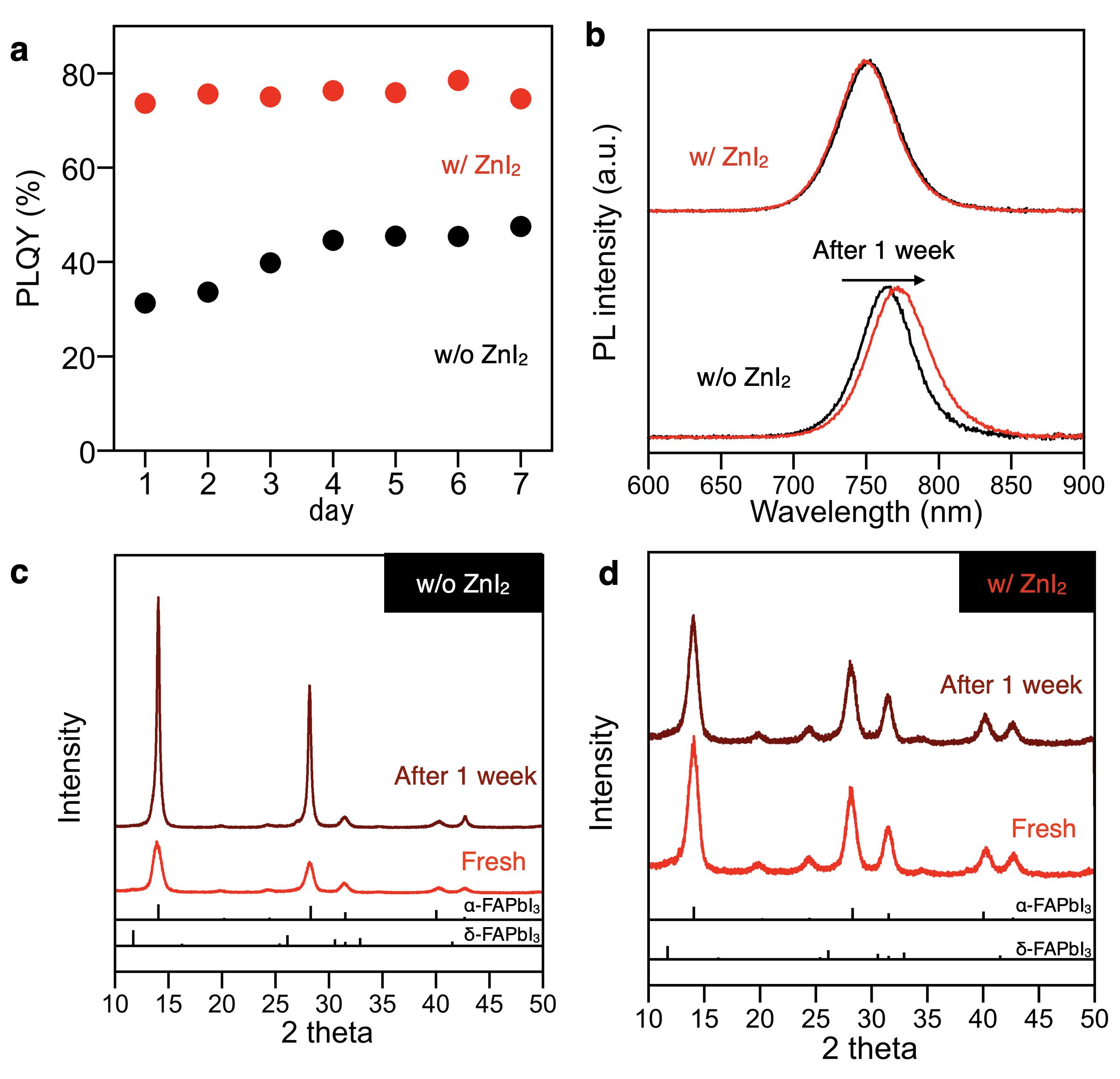
 Figure S2** Stability characterization of FAPbI_3_ NC films with and without ZnI_2_-treatment. (a) Time-dependent PLQY measurements indicating stability with and without ZnI_2_-treatment. (b) Time-dependent PL spectra. XRD pattern of FAPbI_3_ NC films (c) without ZnI_2_-treatment and (d) with ZnI_2_-treatment over time.


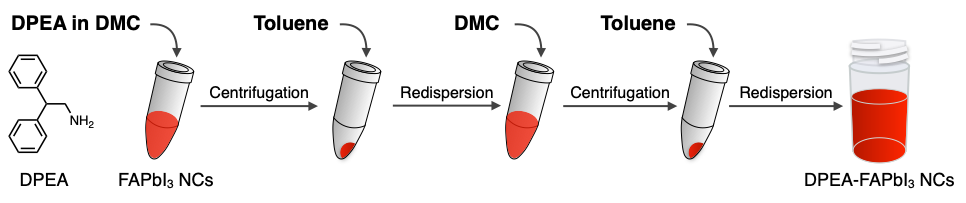


**Figure S3** Ligand exchange and purification procedures of the FAPbI_3_ NCs.


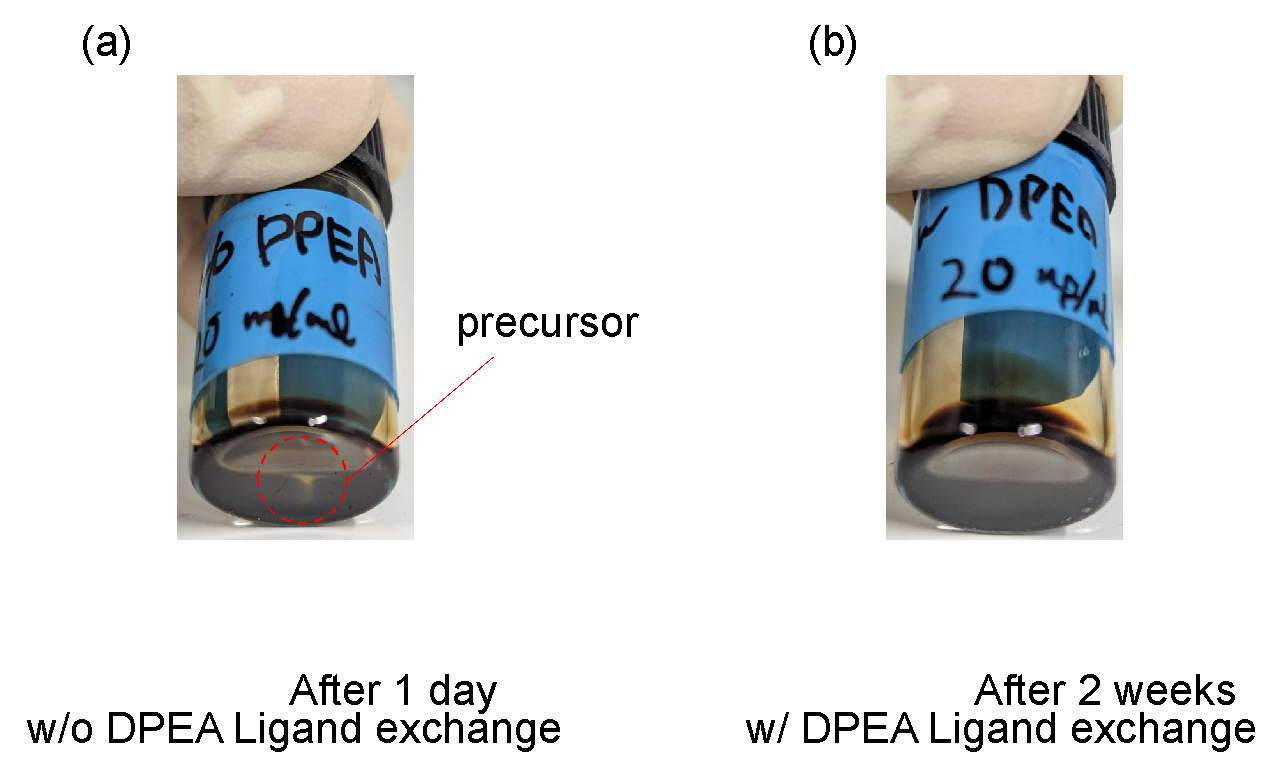


**Figure S4** Colloidal ink stability. (a) FAPbI_3_ NCs and (b) DPEA-FAPbI_3_ NCs in toluene dispersion.


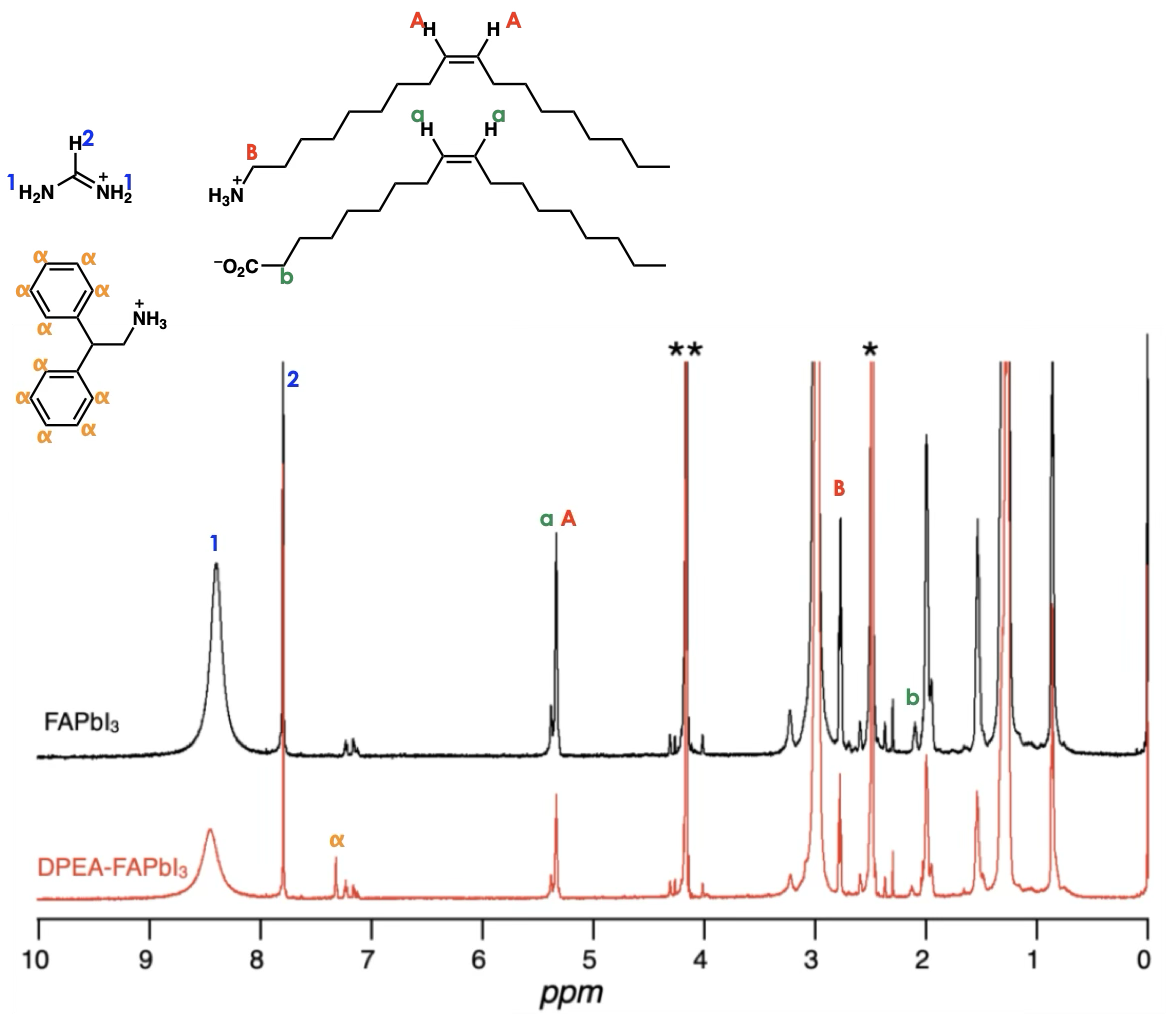


**Figure S5** ^1^HNMR spectra of the FAPbI_3_ NCs and DPEA-FAPbI_3_ NCs in DMSO-*d_6_*. *DMSO and **ferrocene as an internal standard (4.48 × 10^–3^ mol/L for FAPbI_3_ NCs sample and 5.08 × 10^–3^ mol/L for DPEA-FAPbI_3_ NCs sample).


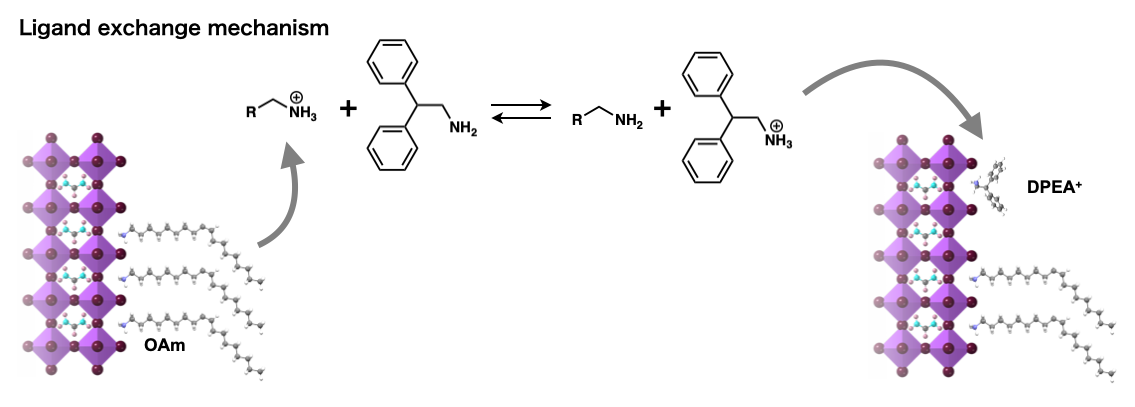


**Figure S6** Ligand exchange scheme between oleylammonium (OAm⁺) and diphenylethylamine (DPEA). The neutral DPEA molecule (–NH₂) undergoes proton transfer with surface-bound OAm⁺ on FAPbI₃ nanocrystals, forming diphenylethylammonium (DPEA⁺), which then replaces OAm⁺ as the surface capping ligand. This exchange enables stable surface modification without introducing excess ionic or structural defects.


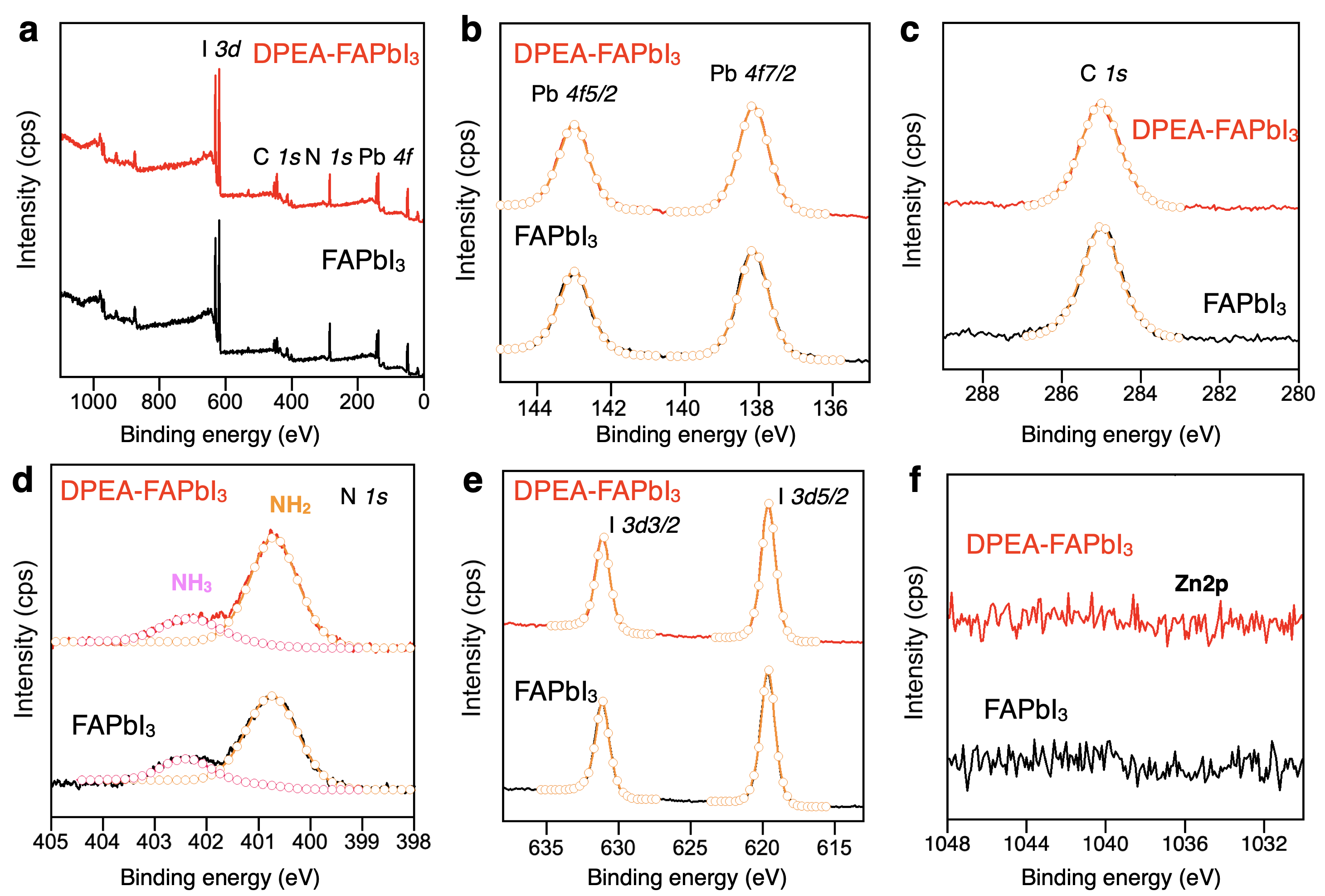


**Figure S7** XPS characterization of FAPbI_3_ NCs before and after DPEA ligand exchange. (a) XPS survey spectra, (b) core spectra of Pb *4f*, (c) core spectra of C *1s*, (d) core spectra of N *1s*, (e) core spectra of I *3d*, and (f) core spectra of Zn *2p*.


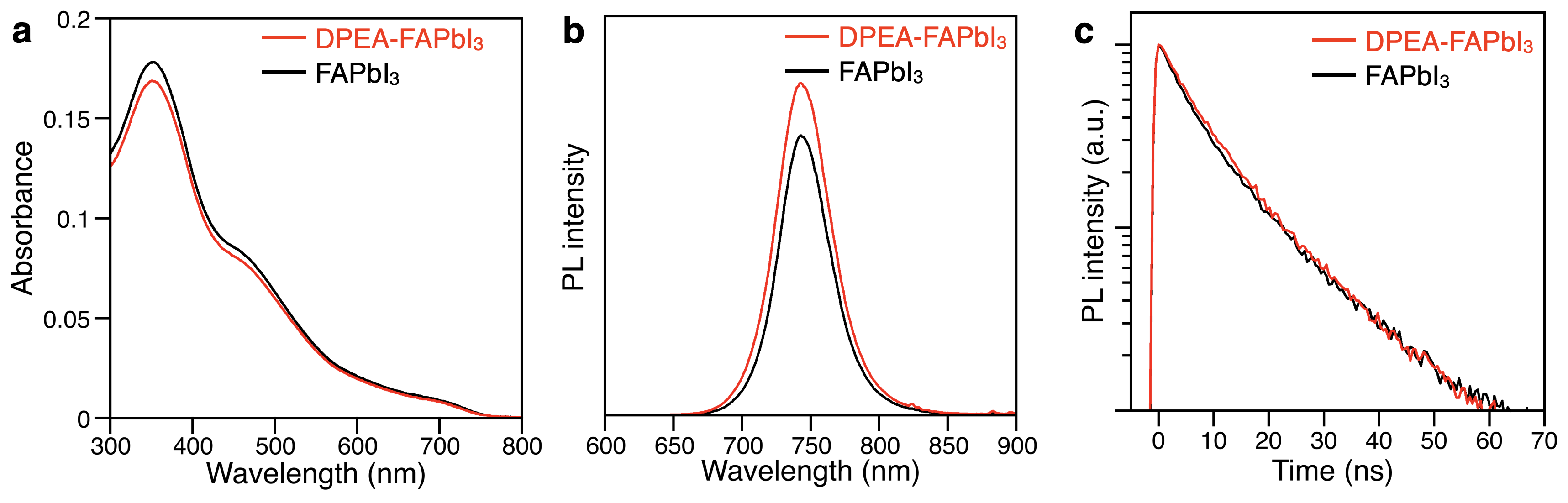


**Figure S8** Optical properties of FAPbI_3_ NCs before and after DPEA ligand exchange. (a) UV-vis absorption spectra, (b) PL spectra, and (c) transient PL decay lifetime of the FAPbI_3_ NC film and DPEA-FAPbI_3_ NC film.


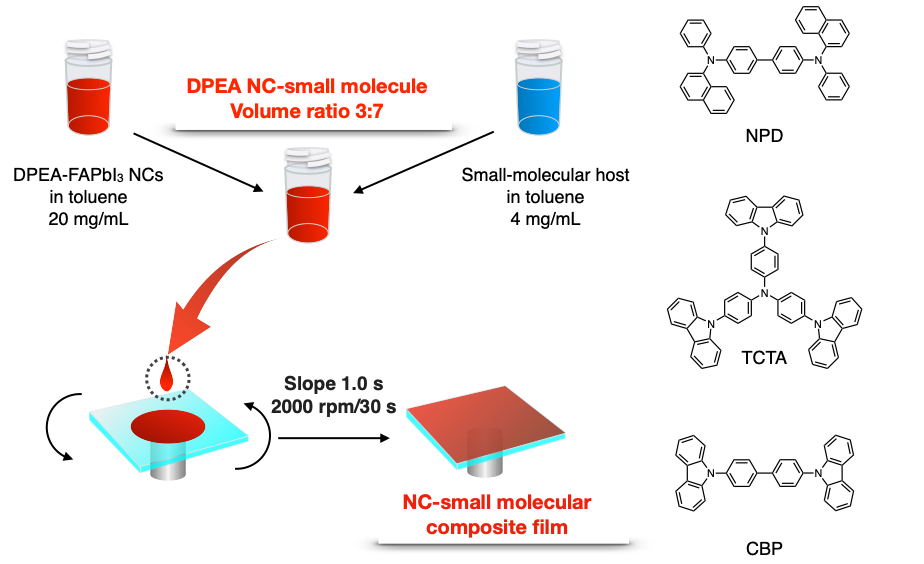


**Figure S9** Preparation process of DPEA-FAPbI_3_ NCs dispersed into organic host materials.

**Figure S10** UV-vis absorption spectra of small-molecule materials: NPD, TCTA, CBP (concentration of 10^–5^ M in toluene).


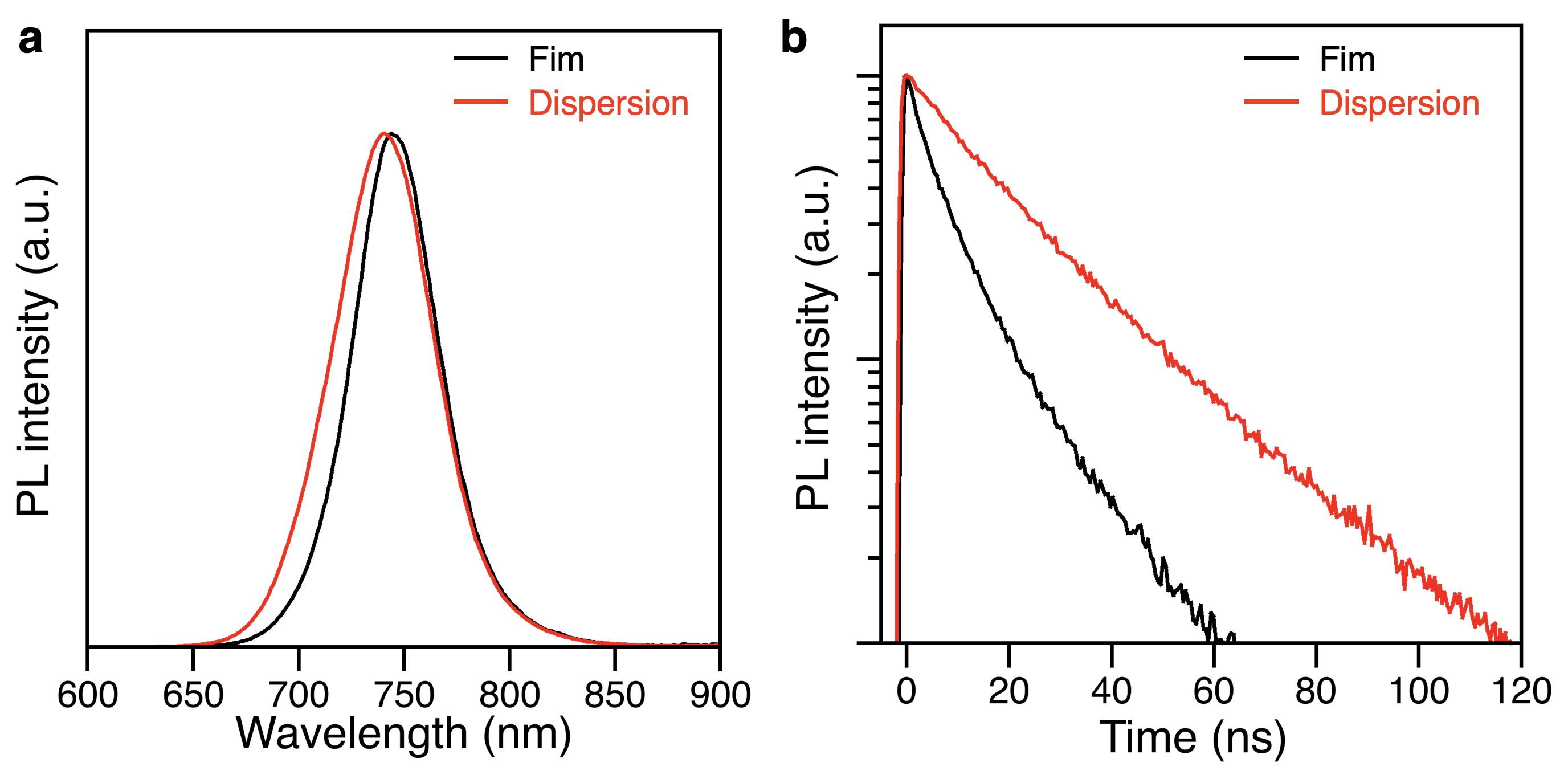


**Figure S11** Optical properties of DPEA-FAPbI_3_ NCs in toluene dispersion and film. (a) PL spectra and (b) transient PL decay lifetime.


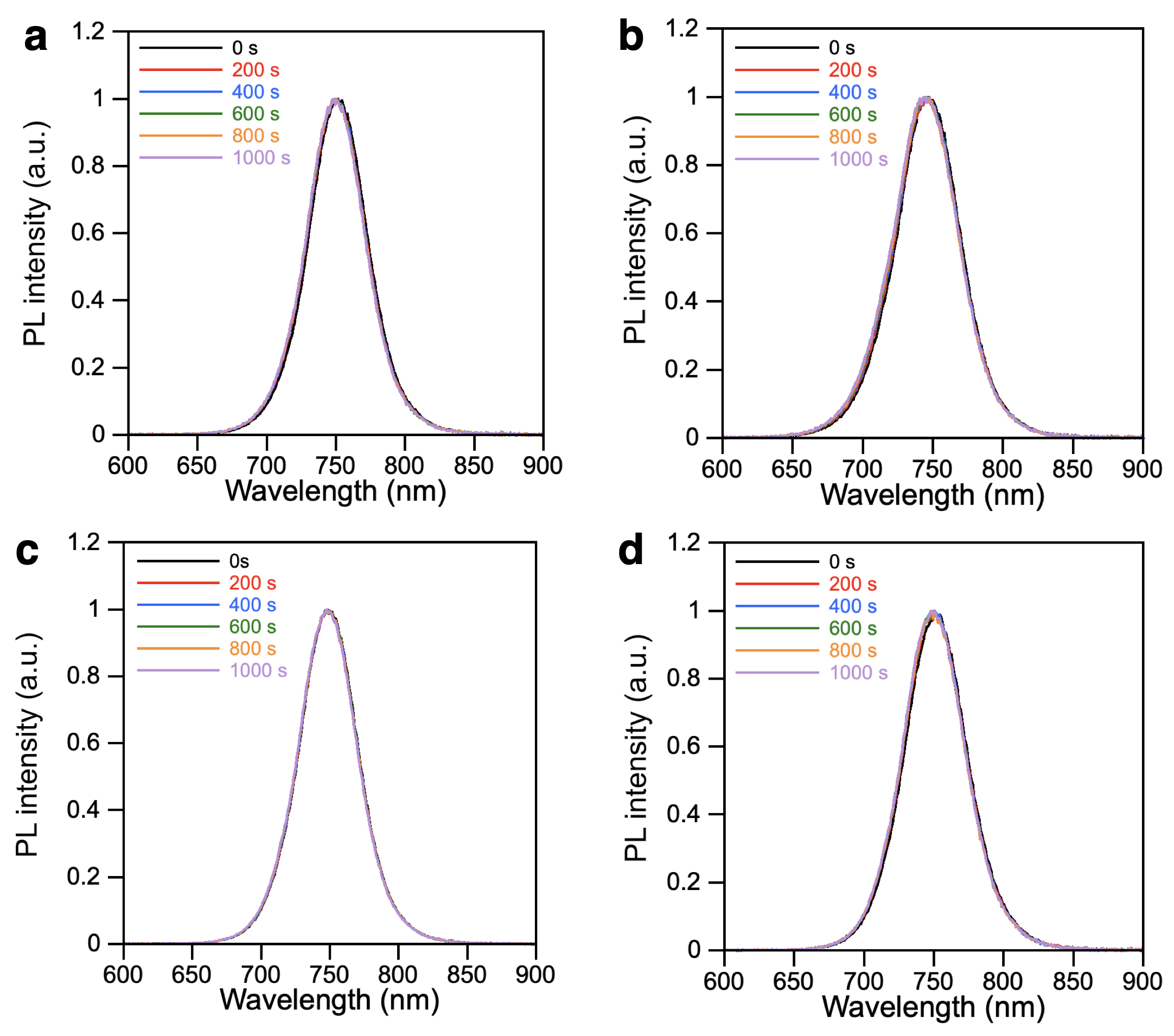


**Figure S12** PL spectra of the organic host-dispersed DPEA-FAPbI_3_ NC films. (a) neat NC film, (b) NPD, (c) TCTA, (d) CBP irradiated for 1000 sec at excitation wavelength 405 nm with power densities of 1.0 mW cm^–2^.


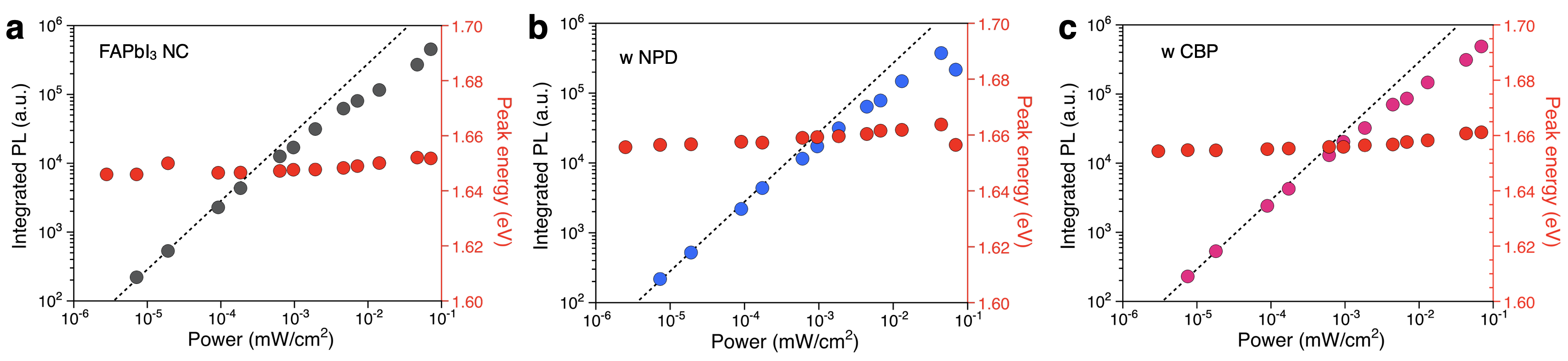


**Figure S13** Integrated PL intensity and emission peak energy as a function of excitation power density for (a) neat FAPbI₃ NC film, (b) NPD-, and (c) CBP-dispersed NC film.


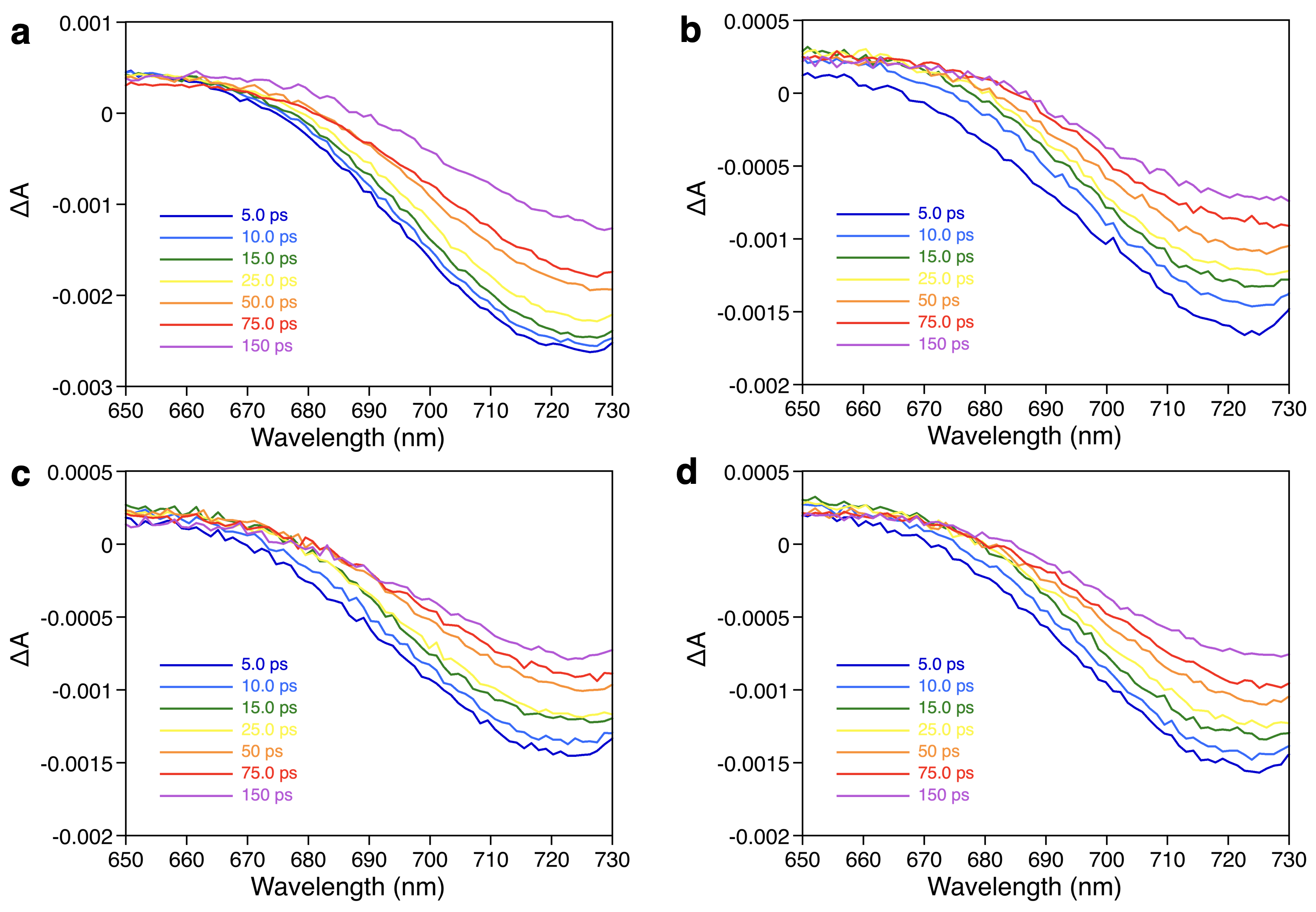


**Figure S14** Femtosecond transient absorption measurements of organic host-dispersed NC films. a: neat DPEA-FAPbI_3_ NC film, b: NPD, c: TCTA, d: CBP.

**
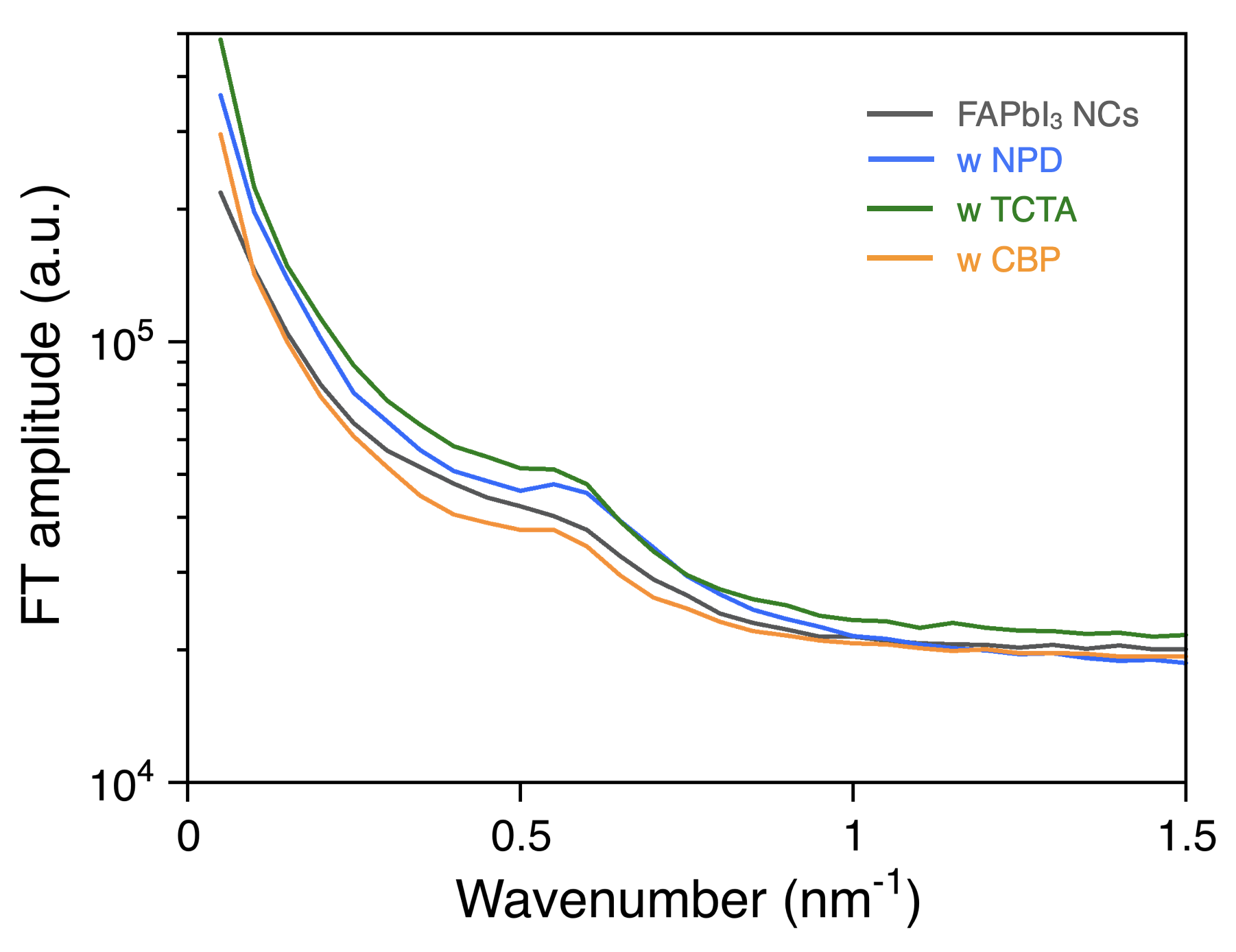
**

**Figure S15** One-dimensional profile converted from the FFT images of organic host-dispersed NC films (NPD, TCTA, and CBP).


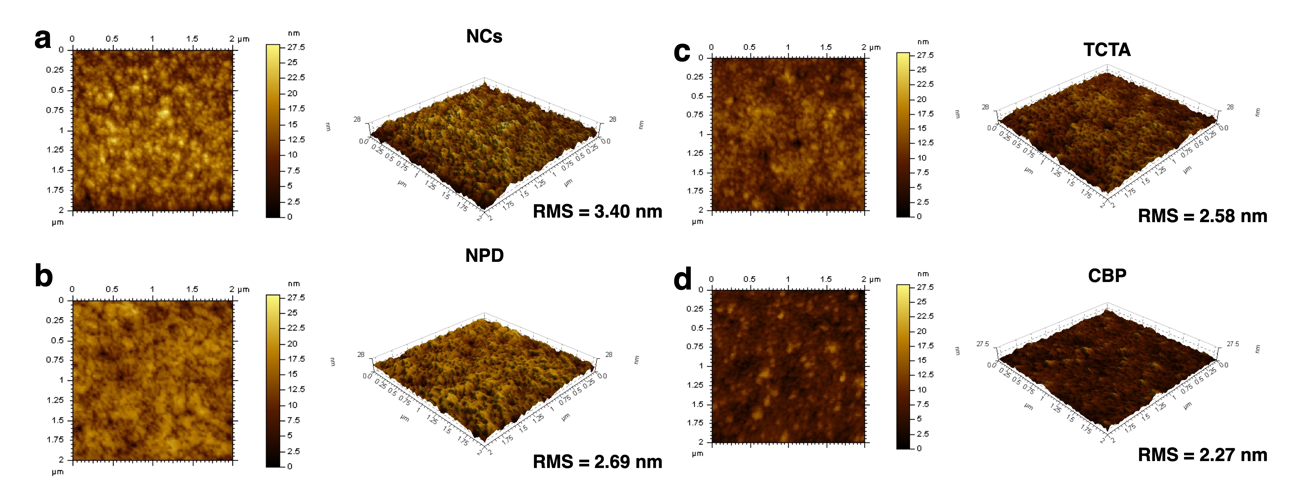


**Figure S16** AFM images of organic host-dispersed NC films. a: neat DPEA-FAPbI_3_ NC film, b: NPD, c: TCTA, d: CBP.

**
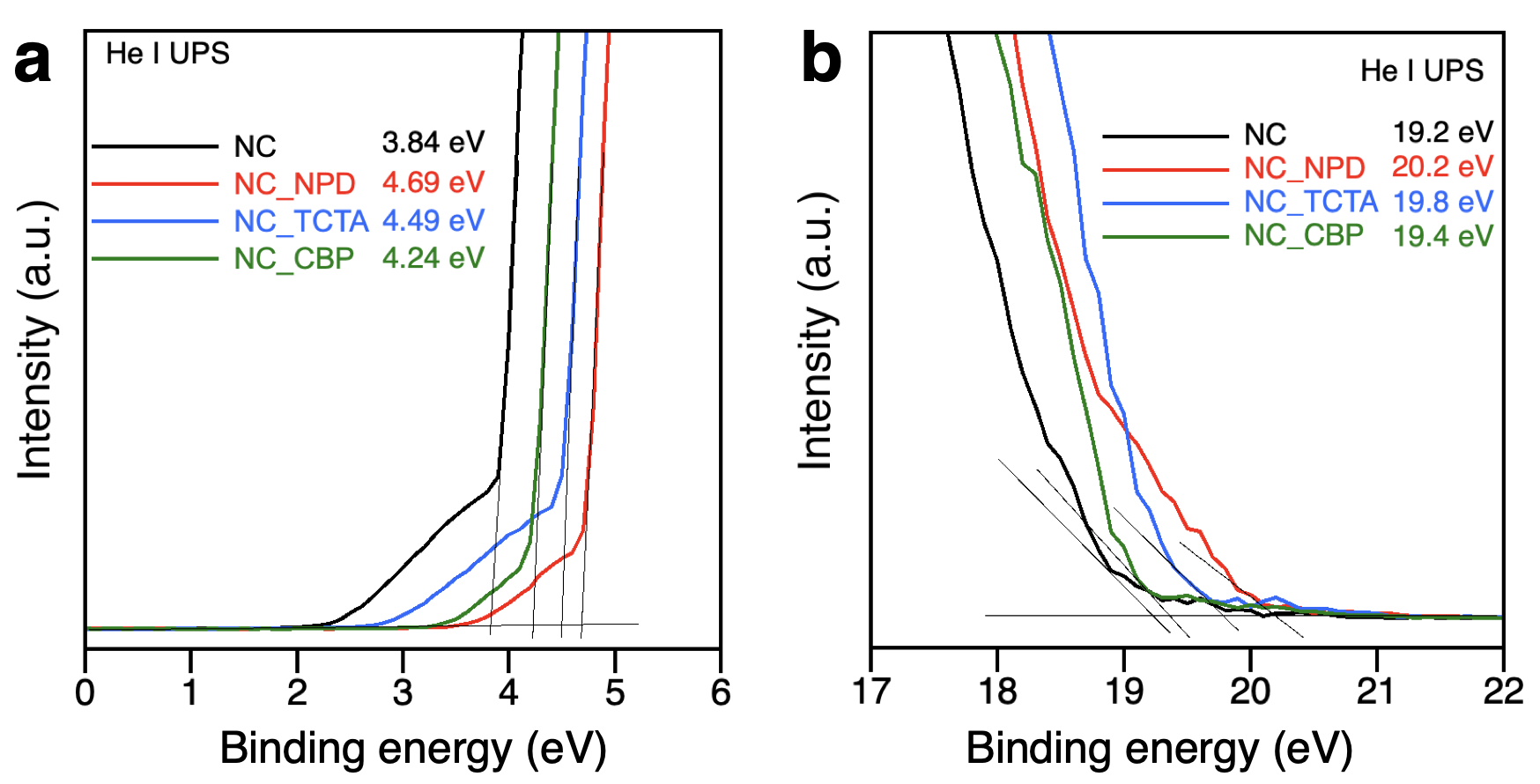
**

**Figure S17** UPS spectra of organic host-dispersed NC films (NPD, TCTA, and CBP).


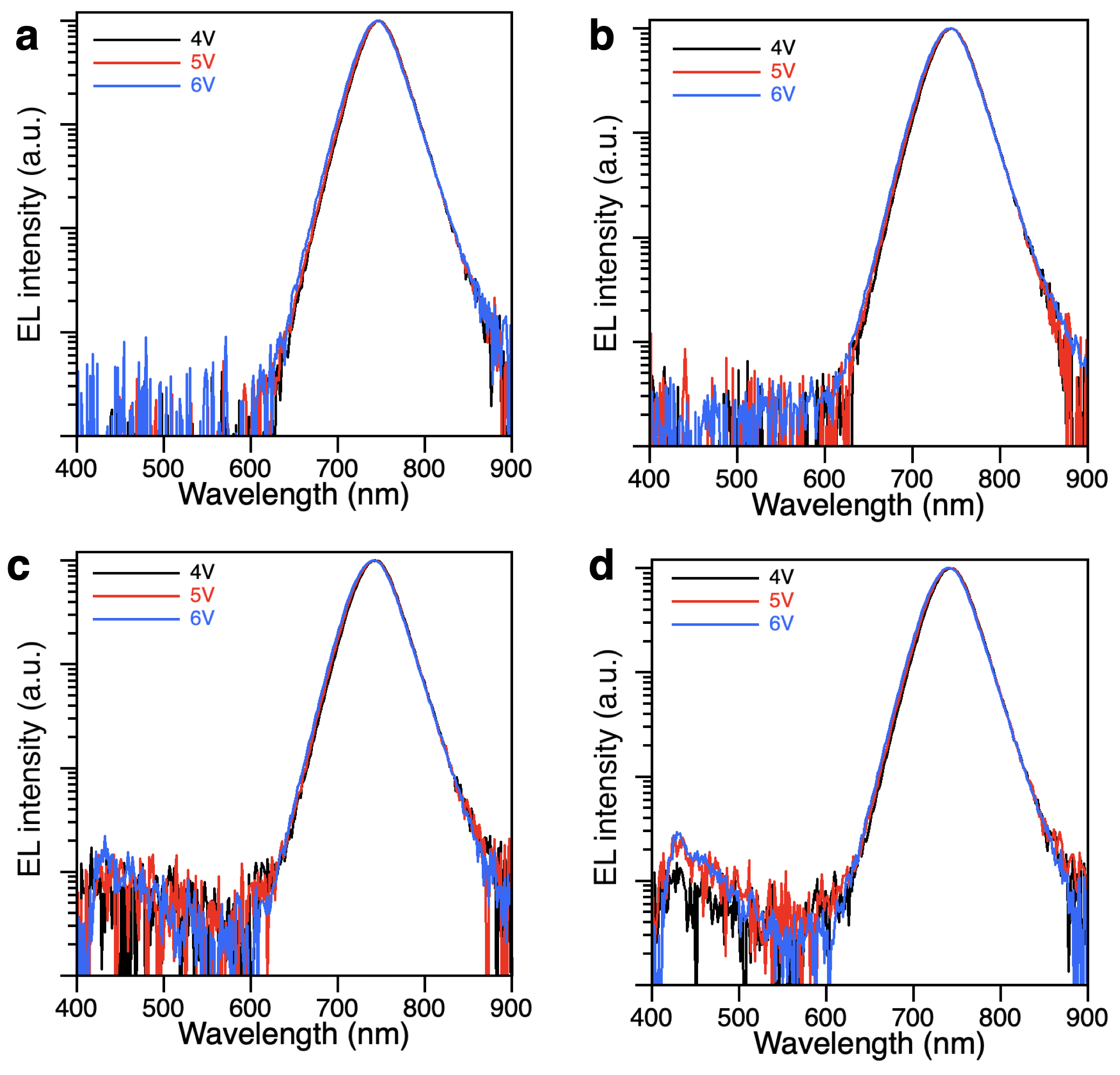


**Figure S18** EL spectra of the LEDs incorporating DPEA-FAPbI_3_ NCs with various organic host materials; a: neat DPEA-FAPbI_3_ NCs, b: NPD, c: TCTA, d: CBP.

**Table S1** XPS characterization of FAPbI_3_ NCs before and after DPEA ligand exchange.

|  | C*1s* | Pb*4f* | I*3d* | N*1s* | I/Pb |
| --- | --- | --- | --- | --- | --- |
| FAPbI_3_ NCs  (w/o ZnI_2_) | 52.94 | 10.17 | 29.21 | 7.68 | 2.87 |
| FAPbI_3_ NCs  (w ZnI_2_) | 67.37 | 6.86 | 20.88 | 6.86 | 3.04 |
| DPEA-NCs | 63.88 | 7.36 | 22.26 | 6.50 | 3.02 |

**Table S2** Concentration of FA, OAm, and DPEA determined from the integral ratio of proton signals (2, B, and α for FA, OAm, and DPEA, respectively) shown in Figure S5. The values in parentheses represent the relative ratios of each species with respect to FA. These results indicate that 4.4% (0.017/0.38) of OAm was ligand exchanged to DPEA.

|  | FA | OAm | DPEA | Ferrocene |
| --- | --- | --- | --- | --- |
| FAPbI_3_ NCs  (w ZnI_2_) | 6.43 × 10^–3^ mol/L  (1) | 2.44 × 10^–3^ mol/L  (0.38) | – | 4.48 × 10^–3^ mol/L  (0.70) |
| DPEA-FAPbI_3_ NCs  (w ZnI_2_) | 6.36 × 10^–3^ mol/L  (1) | 2.00 × 10^–3^ mol/L  (0.31) | 0.11 × 10^–3^ mol/L  (0.017) | 5.08 × 10^–3^ mol/L  (0.79) |

**Table S3** Size and photophysical properties of FAPbI_3_ NC films with host materials.

|  | Size (nm) | PL (nm) | PLQY (%) | τ_PL_ (ns) | *k_r_* (ns^–1^) | *knr* (ns^–1^) |
| --- | --- | --- | --- | --- | --- | --- |
| FAPbI_3_ NCs (wo ZnI_2_) | 10.6 | 756 | 31.3 | 22.2 | 0.014 | 0.031 |
| FAPbI_3_ NCs  (w ZnI_2_) | 10.1 | 744 | 77.8 | 11.0 | 0.070 | 0.020 |
| DPEA-NCs | 10.1 | 744 | 78.4 | 11.3 | 0.069 | 0.019 |
| NPD | 9.7 | 741 | 81.7 | 12.1 | 0.067 | 0.015 |
| TCTA | 10.0 | 741 | 87.5 | 15.6 | 0.056 | 0.008 |
| CBP | 10.1 | 741 | 86.3 | 14.3 | 0.060 | 0.010 |

**Table S4** Summary of device performance of FAPbI_3_ NC-LEDs.

| Publication date | Type | EL peak (nm) | Radiance  (W sr^–1^ m^–2^) | Peak EQE (%) | Device lifetime (LT_50_) | Ref |
| --- | --- | --- | --- | --- | --- | --- |
| 2017.2 | FAPbI_3_ NCs | 772 | 1.54 | 2.3 | NA | 1 |
| 2018.5 | FACsPb(Br/I)_3_ NCs | 735 | 3.9 | 5.9 | 30s  (0.05 W sr^–1^ m^–2^) | 2 |
| 2019.4 | Sn^2+^:FAPbI_3_ NCs | 798 | 0.1 | 1.7 | 35s  (0.1 W sr^–1^ m^–2^) | 3 |
| 2022.3 | PEAI-FAPbI_3_ NCs | 770 | *128.1  (mW cm^–2^) | 15.4 | 15min  (0.1 mW cm^–2^) | 4 |
| 2024.6 | FAPbI_3_ NCs | 778 | *33.4  (mW cm^–2^) | 11.22 | NA | 5 |
| - | DPEA-NCs | 746 | 17.7 | 8.5 | 0.4h  (1.2 W sr^–1^ m^–2^) | This work |
| - | NC:NPD | 742 | 24.7 | 9.6 | 4.5h  (1.2 W sr^–1^ m^–2^) | This work |
| - | NC:TCTA | 742 | 39.9 | 11.1 | 5.2h  (1.2 W sr^–1^ m^–2^) | This work |
| - | NC:CBP | 742 | 26.3 | 14.2 | 1.5h  (1.2 W sr^–1^ m^–2^) | This work |

* Radiance values originally reported in mW cm⁻². These have not been converted to W sr⁻¹ m⁻² to maintain consistency with the original source.

**Table S5** Summary of device performance of FAPbI_3_ based NIR-LEDs.

| Publication date | Type | EL peak (nm) | Radiance  (W s^–1^ rm^–2^) | Peak EQE (%) | Device lifetime (LT_50_) | Ref |
| --- | --- | --- | --- | --- | --- | --- |
| 2016. 6 | Quasi-2D  (PEA)_2_(MA)_n_Pb_n_I_3n+1_ | 758 | 80 | 8.8 | NA | 6 |
| 2016. 9 | Quasi-2D  (NMA)_2_(FAPbI_3_) | 763 | 82 | 11.7 | 2h  (10 mA cm^–2^) | 7 |
| 2017.1 | 3D bulk  BAI-MAPbI_3_ | 748 | 50 cd m^–2^ | 10.4 | 5h  (3mA cm^–2^) | 8 |
| 2018.10 | 3D bulk  5AVA-FAPbI_3_ | 803 | 390 | 20.7 | 20h  (L_0_=100 W sr^–1^ m^–2^) | 9 |
| 2018.11 | 2D/3D bulk | 795 | <10 | 20.1 | 46h  (L_0_=0.1 W sr^–1^ m^–2^) | 10 |
| 2019.3 | 3D bulk  ODEA-FAPbI_3_ | 800 | 308 | 21.6 | 20h  (L_0_=20 W sr^–1^ m^–2^) | 11 |
| 2019.8 | Quasi-2D  BAB-FAPbI_3_ | 776 | 88.5 | 5.2 | 100h  (L_0_=35 W sr^–1^ m^–2^) | 12 |
| 2020.2 | 3D bulk  DDS-FAPbI_3_ | 802 | <100 | 17.3 | 120h  (L_0_=15 W sr^–1^ m^–2^) | 13 |
| 2021.4 | 3D bulk  3ClBA-FAPbI_3_ | 798 | 300 | 16.6 | 49h  (L_0_=100 W sr^–1^ m^–2^) | 14 |
| 2023.3 | 3D bulk  MSPE-FAPbI_3_ | 800 | 3200 | 23.8 | 32h  (L_0_=100 W sr^–1^ m^–2^) | 15 |
| 2023.12 | 3D bulk  5AVAI-FAPbI_3_ | 800 | 207 | 15.1 | NA | 16 |
| 2024.5 | 3D bulk  5AVA-PyNI-FAPbI_3_ | 800 | 390 | 32.0 | 17h  (L_0_=100 W sr^–1^ m^–2^) | 17 |
| 2024.10 | 3D bulk  FAPbI_3_/ZnMgO | 802 | 648 | 22.3 | 1400sec  (L_0_=100 W sr^–1^ m^–2^) | 18 |

**References**

[1] L. Protesescu, S. Yakunin, S. Kumar, J. Bär, F. Bertolotti, N. Masciocchi, A. Guagliardi, M. Grotevent, I. Shorubalko, M. I. Bodnarchuk, C. J. Shih, M. V. Kovalenko, *ACS Nano* **2017**, 11, 3119.

[2] I. Lignos, V. Morad, Y. Shynkarenko, C. Bernasconi, R. M. Maceiczyk, L. Protesescu, F. Bertolotti, S. Kumar, S. T. Ochsenbein, N. Masciocchi, A. Guagliardi, C. J. Shih, M. I. Bodnarchuk, A. J. deMello, M. V. Kovalenko, *ACS Nano* **2018**, 12, 5504.

[3] R. Begum, X. Y. Chin, M. J. Li, B. Damodaran, T. C. Sum, S. Mhaisalkar, N. Mathews, *Chem. Commun.* **2019**, 55, 5451.

[4] Z. L. Tseng, L. C. Chen, L. W. Chao, M. J. Tsai, D. A. Luo, N. R. Al Amin, S. W. Liu, K. T. Wong, *Adv. Mater.* **2022**, 34, 2109785.

[5] Z. L. Tseng, S. A. Chen, J. H. Lin, K. Y. Ke, K. Uma, *Ceram. Int.* **2024**, 50, 35257.

[6] M. J. Yuan, L. N. Quan, R. Comin, G. Walters, R. Sabatini, O. Voznyy, S. Hoogland, Y. B. Zhao, E. M. Beauregard, P. Kanjanaboos, Z. H. Lu, D. H. Kim, E. H. Sargent, *Nat. Nanotechnol.* **2016**, 11, 872.

[7] N. N. Wang, L. Cheng, R. Ge, S. T. Zhang, Y. F. Miao, W. Zou, C. Yi, Y. Sun, Y. Cao, R. Yang, Y. Q. Wei, Q. Guo, Y. Ke, M. T. Yu, Y. Z. Jin, Y. Liu, Q. Q. Ding, D. W. Di, L. Yang, G. C. Xing, H. Tian, C. H. Jin, F. Gao, R. H. Friend, J. P. Wang, W. Huang, *Nat. Photonics.* **2016**, 10, 699.

[8] Z. G. Xiao, R. A. Kerner, L. F. Zhao, N. L. Tran, K. M. Lee, T. W. Koh, G. D. Scholes, B. P. Rand, *Nat. Photonics.* **2017**, 11, 108.

[9] Y. Cao, N. N. Wang, H. Tian, J. S. Guo, Y. Q. Wei, H. Chen, Y. F. Miao, W. Zou, K. Pan, Y. R. He, H. Cao, Y. Ke, M. M. Xu, Y. Wang, M. Yang, K. Du, Z. W. Fu, D. C. Kong, D. X. Dai, Y. Z. Jin, G. Q. Li, H. Li, Q. M. Peng, J. P. Wang, W. Huang, *Nature* **2018**, 562, 249.

[10] B. D. Zhao, S. Bai, V. Kim, R. Lamboll, R. Shivanna, F. Auras, J. M. Richter, L. Yang, L. J. Dai, M. Alsari, X. J. She, L. S. Liang, J. B. Zhang, S. Lilliu, P. Gao, H. J. Snaith, J. P. Wang, N. C. Greenham, R. H. Friend, D. W. Di, *Nat. Photonics.* **2018**, 12, 783.

[11] W. D. Xu, Q. Hu, S. Bai, C. X. Bao, Y. F. Miao, Z. C. Yuan, T. Borzda, A. J. Barker, E. Tyukalova, Z. J. Hu, M. Kawecki, H. Y. Wang, Z. B. Yan, X. J. Liu, X. B. Shi, K. Uvdal, M. Fahlman, W. J. Zhang, M. Duchamp, J. M. Liu, A. Petrozza, J. P. Wang, L. M. Liu, W. Huang, F. Gao, *Nat. Photonics.* **2019**, 13, 418.

[12] Y. Q. Shang, Y. Liao, Q. Wei, Z. Y. Wang, B. Xiang, Y. Q. Ke, W. M. Liu, Z. J. Ning, *Sci. Adv.* **2019**, 5, eaaw8072.

[13] H. Y. Wang, F. U. Kosasih, H. L. Yu, G. H. J. Zheng, J. B. Zhang, G. Pozina, Y. Liu, C. X. Bao, Z. J. Hu, X. J. Liu, L. Kobera, S. Abbrent, J. Brus, Y. Z. Jin, M. Fahlman, R. H. Friend, C. Ducati, X. K. Liu, F. Gao, *Nat. Commun.* **2020**, 11, 891.

[14] H. Zhang, C. L. Tu, C. Xue, J. H. Wu, Y. Cao, W. Zou, W. J. Xu, K. C. A. Wen, J. Zhang, Y. Chen, J. Y. Lai, L. Zhu, K. Pan, L. Xu, Y. Q. Wei, H. Z. Lin, N. N. Wang, W. Huang, J. P. Wang, *Nano Lett.* **2021**, 21, 3738.

[15] Y. Q. Sun, L. S. Ge, L. J. Dai, C. S. Cho, J. F. Orri, K. Y. Ji, S. J. Zelewski, Y. Liu, A. J. Mirabelli, Y. C. Zhang, J. Y. Huang, Y. S. Wang, K. Gong, M. C. Lai, L. Zhang, D. Yang, J. D. Lin, E. M. Tennyson, C. Ducati, S. D. Stranks, L. S. Cui, N. C. Greenham, *Nature* **2023**, 615, 830.

[16] J. Jiménez‐López, D. Cortecchia, E. L. Wong, G. Folpini, A. Treglia, A. L. Alvarado‐Leaños, C. S. Wu, A. Olivati, A. Petrozza, *Adv. Funct. Mater.* **2023**, DOI: 10.1002/adfm.2023085452308545.

[17] M. M. Li, Y. G. Yang, Z. Y. Kuang, C. J. Hao, S. X. Wang, F. Y. Lu, Z. R. Liu, J. L. Liu, L. J. Zeng, Y. X. Cai, Y. L. Mao, J. S. Guo, H. Tian, G. C. Xing, Y. Cao, C. Ma, N. N. Wang, Q. M. Peng, L. Zhu, W. Huang, J. P. Wang, *Nature* **2024**, 630, 631.

[18] X. Y. Yang, Y. Q. Ji, Q. Y. Li, Q. X. Zhong, H. Li, Z. Y. C. Lu, H. H. Chen, Y. J. Wang, A. Hu, S. D. Li, L. Ma, L. Li, Y. Z. Zhang, Y. Chen, L. C. Zhao, J. Wu, X. Q. Wang, C. J. Lu, R. Zhu, *Adv. Funct. Mater.* **2024**, DOI: 10.1002/adfm.2024135172413517.
